# A Survey on Intelligent Perception, State Estimation and Multimodal Navigation Technologies for Resource-Constrained Autonomous Systems

**DOI:** 10.3390/s26216728

**Published:** 2026-10-24

**Authors:** Peng Jiang, Heng Zheng, Yongqiang Han, Lina Ju, Zhengui Huang, Yifan Zhao

**Affiliations:** 1 National Key Laboratory of Transient Physics, Nanjing University of Science and Technology, Nanjing 210094, China; jpswdtc0723@njust.edu.cn; 2 East China Institute of Photo Electron, Suzhou 215163, China; 18015550920@126.com; 3 School of Instrument Science and Technology, Xi’an Jiaotong University, Xi’an 710049, China; zhengheng666@stu.xjtu.edu.cn; 4 School of Automation, Beijing Institute of Technology, Beijing 100081, China; hanyongqiang213@163.com; 5 Faculty of Marine Science and Technology, Beijing Institute of Technology, Zhuhai 519088, China

**Keywords:** resource-constrained autonomous systems, intelligent perception, learning-enhanced state estimation, multimodal sensor fusion, edge AI navigation

## Abstract

Resource-constrained autonomous systems, including small UAVs, mobile robots, and industrial AGVs, must achieve reliable navigation under strict limits on computation, power, memory, payload, and communication. This survey reviews four connected areas: intelligent perception, learning-enhanced state estimation, multimodal navigation fusion, and edge deployment. It analyzes how recent methods balance accuracy, robustness, latency, and energy efficiency on embedded platforms. The synthesis shows a shift from isolated algorithm optimization toward integrated, deployment-aware navigation frameworks and identifies remaining challenges in generalization, benchmarking, and system-level validation.

## 1. Introduction

Autonomous systems increasingly operate in dynamic, unstructured, and GNSS-denied environments. Reliable navigation therefore requires real-time perception, state estimation, planning, and control under limited computation and power. In addition to accurate, low-drift pose estimates, the platform must recognize semantic, geometric, and dynamic properties of its surroundings to support safe and sustained operation.

These systems face heterogeneous sensing, nonlinear motion, uncertain environments, and strict resource limits. Cameras provide rich semantics but degrade under poor illumination, blur, occlusion, weather, or weak texture. LiDAR supplies metric geometry but adds cost, weight, power, and processing load; IMUs are fast and efficient but drift; and GNSS is globally referenced but vulnerable to blockage and multipath. Asynchronous sampling, calibration drift, dynamic objects, and non-Gaussian errors further complicate fusion, while small platforms cannot simply absorb these difficulties with larger networks or denser optimization.

**Figure 1:**
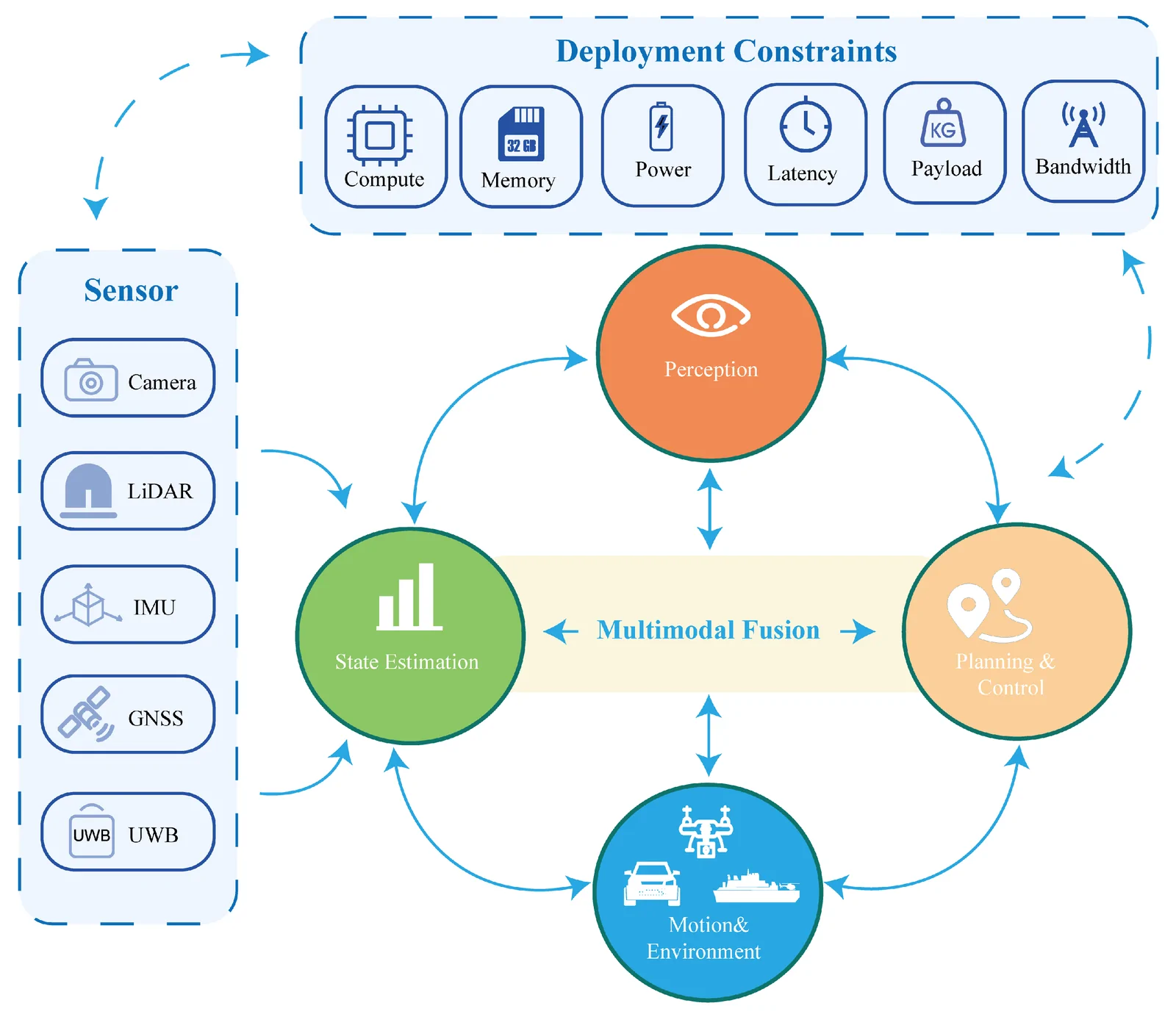
Organizational framework used in this review.

As shown in Figure 1, the proposed framework places intelligent perception and state estimation within a closed navigation loop that connects sensor observations, planning and control, and platform–environment interaction. Multimodal fusion serves as a cross-cutting information-integration mechanism, while deployment constraints apply across the entire loop. The figure therefore illustrates the functional relationships among these components rather than a fixed sequential processing pipeline.

Classical navigation relies on geometric modeling, probabilistic filters, visual or LiDAR odometry, inertial preintegration, and factor-graph optimization. These methods remain interpretable and effective in well-modeled conditions, but fixed noise assumptions, handcrafted features, calibration sensitivity, and expensive optimization can reduce robustness or deployability when sensing conditions and platform resources change.

AI has expanded this foundation through learned perception, neural filtering, differentiable optimization, reinforcement learning, and edge-oriented model compression. Deep networks support object and scene understanding, BEV and occupancy representations; learning modules can adapt noise models or estimator updates; and multimodal methods increasingly align visual, LiDAR, inertial, GNSS, radar, UWB, and event data in shared representations or optimization frameworks. Lightweight networks and hardware acceleration seek to make these capabilities practical onboard.

Table 1 provides a scope-based comparison of representative reviews. Several previous surveys cover more than one component of autonomous navigation, but they primarily emphasize path planning, localization, scene understanding, or sensor fusion. The contribution of this survey lies in organizing intelligent perception, learning-enhanced state estimation, multimodal fusion, and edge deployment within a common framework centered on resource-constrained operation. This comparison describes differences in thematic scope and does not imply that the present survey is methodologically superior to the existing reviews.

This paper reviews AI-enabled navigation for resource-constrained autonomous systems, emphasizing how accuracy, robustness, latency, energy use, and deployability interact in closed-loop operation. The main contributions are summarized as follows.

First, this paper organizes the literature around the complete perception–estimation–fusion–deployment loop, enabling the relationships between algorithmic performance and embedded constraints to be examined at the system level.

Second, it critically compares representative perception, learning-enhanced estimation, sensor–fusion, and edge-deployment methods, with particular attention to sensor degradation, calibration, synchronization, computational cost, and practical deployment.

Third, it summarizes failure-aware fusion and uncertainty-handling strategies, including sensor-quality assessment, adaptive weighting, outlier rejection, fallback operation, and safe degradation.

Fourth, it compares commonly used datasets and evaluation metrics and identifies open challenges in cross-platform generalization, long-term robustness, computational efficiency, and reproducible deployment-oriented evaluation.

The remainder of this paper presents the review methodology and taxonomy (Section 2), perception (Section 3), state estimation (Section 4), multimodal fusion (Section 5), edge deployment (Section 6), datasets and evaluation (Section 7), and conclusions (Section 8).

## 2. Review Methodology and Taxonomy

### 2.1. Scope Definition

This review concerns small UAVs, mobile robots, AGVs, agricultural and inspection robots, and similar platforms whose onboard computation, power, payload, thermal capacity, storage, or communication bandwidth constrain algorithm and sensor choices. Despite these limits, they must perform real-time perception, localization, mapping, and navigation in dynamic environments. The review therefore emphasizes trade-offs among accuracy, robustness, latency, and deployability.

The scope covers four interrelated topics: navigation-oriented perception, including 2D–3D, Camera–LiDAR, BEV, and occupancy methods; learning-enhanced state estimation, including adaptive and neural filtering and learning-assisted VIO/SLAM; multimodal fusion using visual, inertial, GNSS, LiDAR, UWB, and factor-graph formulations; and edge deployment through lightweight models, bounded optimization, compression, and hardware acceleration.

It should be noted that this paper does not attempt to cover all research directions related to autonomous systems. Pure path planning, motion control, mechanical structure design, communication protocols, cloud-based large-model training and general-purpose visual recognition tasks that are weakly coupled with the navigation loop are not the main focus of this review. For studies related to autonomous driving, this paper mainly focuses on multimodal perception, state estimation, and edge deployment methods that can be transferred to resource-constrained autonomous systems, rather than providing a comprehensive discussion of complete autonomous driving system architectures.

### 2.2. Literature Collection and Qualitative Selection Strategy

This article is intended as a narrative technical survey rather than a PRISMA-style systematic review. To provide a structured overview of recent advances in intelligent perception, state estimation, and multimodal navigation, relevant literature was collected mainly from Web of Science, IEEE Xplore, ScienceDirect, SpringerLink, ACM Digital Library, Google Scholar, arXiv, and MDPI. Studies were selected qualitatively according to their relevance to autonomous navigation, environmental perception, state estimation, multi-sensor fusion, or edge deployment, with priority given to work presenting clear algorithmic frameworks, system architectures, or experimental validation. Because this selection process was designed to represent the principal technical directions covered by the proposed taxonomy rather than to identify every eligible publication, the reviewed studies should be regarded as representative rather than exhaustive; relevant work outside the selected information sources and technical categories may therefore be underrepresented.

### 2.3. Analytical Framework and Classification Dimensions

The literature is organized along overlapping functional, integration, and implementation dimensions. Perception describes environmental understanding; state estimation describes pose, motion, and uncertainty inference; multimodal fusion describes how heterogeneous observations are combined within either function; and edge deployment describes the resource conditions that constrain all of them. VIO and LIO, for example, are both state-estimation and fusion methods whose design depends on onboard computation, memory, latency, and power.

Accordingly, this review organizes the literature into four interrelated, as the Table 2 shows, non-exclusive analytical dimensions rather than four sequential processing layers. Intelligent perception concerns external environmental understanding, and learning-enhanced state estimation concerns pose, motion, and uncertainty inference. Multimodal navigation fusion is a cross-cutting integration mechanism that combines heterogeneous observations within perception, estimation, mapping, or other navigation functions. Edge-intelligent deployment is a cross-cutting implementation dimension that evaluates whether these functions and mechanisms can operate under limited computation, memory, power, payload, thermal capacity, and communication bandwidth.

This taxonomy supports comparison and does not prescribe a serial architecture. In practice, fused measurements may directly update the state, motion estimates may assist perception, and deployment limits may alter sensor selection, model complexity, update rates, and scheduling throughout the closed navigation loop.

## 3. Intelligent Perception

### 3.1. The Role of Intelligent Sensing in Autonomous Navigation

**Figure 2:**
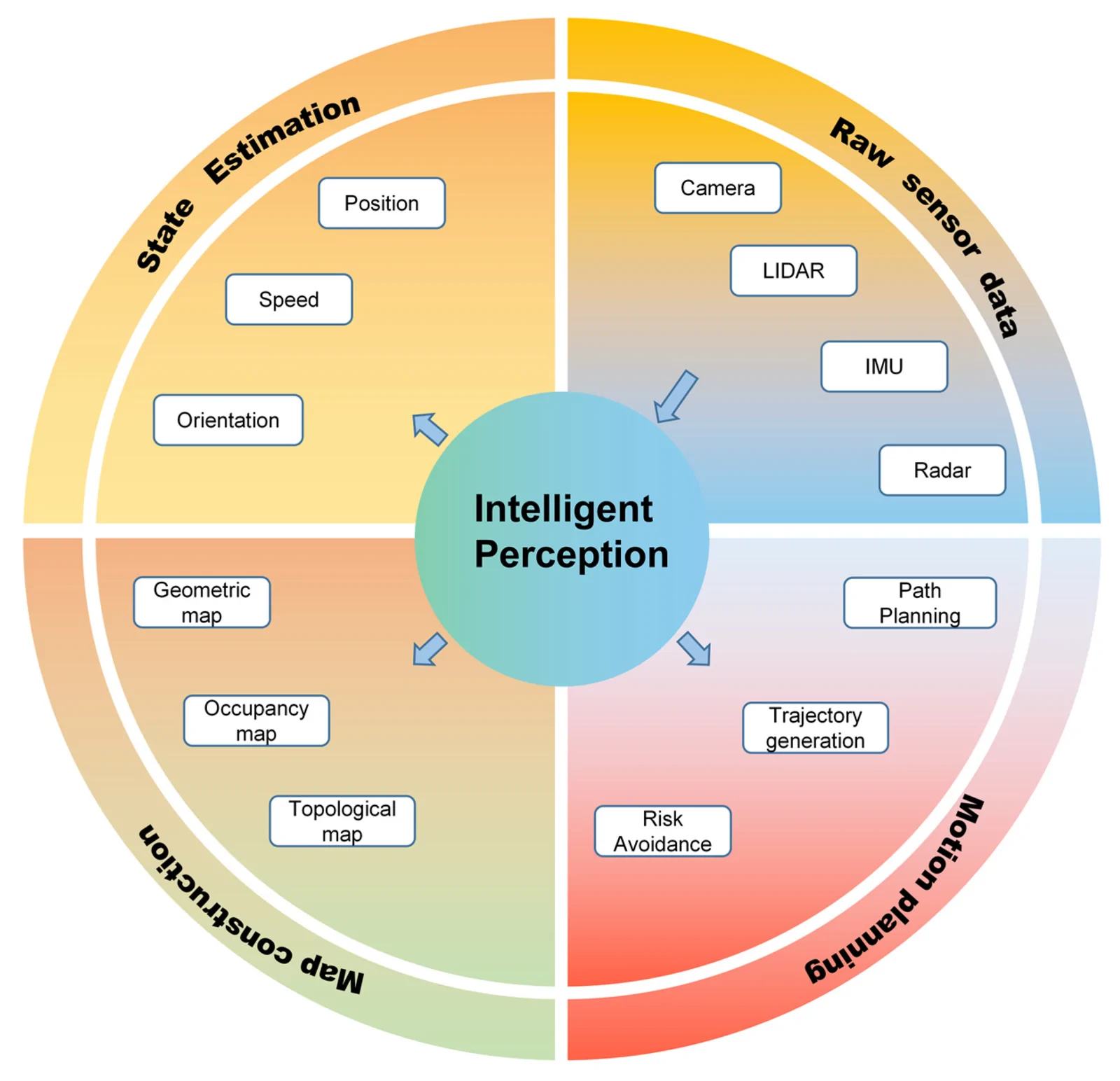
Role of intelligent perception as an intermediate layer in the closed-loop navigation framework of resource-constrained autonomous systems.

Intelligent perception converts sensor data into environmental information for localization, mapping, obstacle avoidance, planning, and task execution [11,12,13,14]. As shown in Figure 2, it links sensing to state estimation, mapping, and planning by answering navigation-relevant questions about surrounding objects, traversability, motion, targets, and structural change [15,16].

Perception outputs range from objects, boundaries, depth, and local features to structured representations such as free space, semantic maps, BEV grids, occupancy, and dynamic-object states [17]. Detection and segmentation identify obstacles or targets, depth and 3D perception support collision checking and mapping, and BEV or occupancy representations provide planning-oriented spatial constraints.

On small platforms, perception must satisfy accuracy, latency, memory, and power limits simultaneously [18,19,20,21,22]. Task examples include high-rate obstacle detection on UAVs, semantic mapping on low-power mobile robots, and target recognition under unstructured field conditions [23,24]. Evaluation should, therefore, include computational cost, memory, energy, and sustained runtime in addition to mAP, mIoU, or accuracy [25,26].

No single sensor covers all conditions [27]. Cameras are inexpensive and semantically rich but sensitive to lighting, blur, occlusion, texture, and weather [28]. LiDAR provides metric geometry at greater cost and payload [29]; depth cameras are range- and lighting-limited; event cameras offer low latency but a less mature toolchain. Learning-based multimodal representations can exploit these complementary properties [30].

In summary, intelligent perception plays the role of transforming raw sensor data into navigation-usable environmental representations in autonomous navigation systems. Its development trend can be summarized as follows: from single-modal perception to multimodal fusion, from two-dimensional detection to three-dimensional scene understanding, from local object recognition to unified spatial representations. Following this logic, the subsequent subsections discuss vision-based task and scene perception, two-dimensional–three-dimensional joint perception, Camera-LiDAR fusion and BEV-occupancy environmental representation methods.

### 3.2. Vision-Based Task and Scene Perception

Vision-based perception is one of the most commonly used environmental perception approaches in resource-constrained autonomous systems [31]. Cameras are particularly suitable for visual perception modules on platforms such as small UAVs, mobile robots, and industrial AGVs because of their low cost, compact size, low power consumption, high information density and ease of integration. Compared with active sensors such as LiDAR and radar, visual sensors can provide rich texture, color, edge and semantic information, enabling autonomous systems to recognize object categories, understand scene structures, detect dynamic obstacles and localize task-related targets. Therefore, in many resource-constrained platforms, vision-based perception often serves as the basic front-end for environmental understanding and task target recognition.

Vision-based perception for navigation tasks is not a single image recognition problem, but a hierarchical process consisting of object recognition, region understanding, traversability estimation and dynamic risk perception. For autonomous systems, the visual front-end first needs to determine what objects are present in the scene, then further understand the semantic attributes and spatial relationships of different regions, and finally transform the perception results into environmental constraints that can be used for obstacle avoidance, path planning and task execution. Therefore, object detection, semantic segmentation, instance segmentation, traversable-area recognition and dynamic object perception support the autonomous navigation loop at the object, pixel, instance and risk levels, respectively [32]. Table 3 summarizes the output of each perception task and its corresponding navigation use.

Object detection is the most fundamental object-level perception task in visual navigation. It is mainly used to rapidly localize key objects in a scene, such as obstacles, pedestrians, vehicles, landmarks, operational targets, landing areas and other task-related objects, outputting their categories and two-dimensional image locations. Such results can provide candidate targets for subsequent object tracking, ranging, obstacle avoidance or task-level decision-making. Compared with traditional detection methods based on color, texture, edges or geometric rules, deep learning-based object detection networks can learn more discriminative visual features under complex backgrounds, scale variations, partial occlusions, and large differences in object appearance. As a result, they have become widely used visual front-ends for platforms such as mobile robots, UAVs, and AGVs [33,34].

Bounding boxes do not describe precise boundaries or traversable space [35,36]. Semantic segmentation supplies pixel-level classes for roads, ground, walls, vegetation, water, and dynamic objects [37,38], while instance segmentation separates individual objects for tracking, avoidance, and target selection [39].

Traversable-area recognition and dynamic-object perception connect vision directly to motion decisions [40,41]. They estimate where the platform can move and how surrounding agents may create collision risk, providing more actionable information than isolated detection or segmentation outputs.

Embedded visual perception requires lightweight models. MobileNet [42], ShuffleNet [43], EfficientDet [44], compact YOLO/Transformer variants, pruning, quantization, and distillation reduce computation through efficient convolutions, feature reuse, and simplified heads. Navigation-oriented designs can further prioritize obstacles, traversable regions, dynamic objects, and task targets instead of modeling every category; lightweight encoders and selective high-resolution processing similarly reduce segmentation cost.

Visual semantic segmentation also requires a lightweight design. High-accuracy segmentation networks usually involve high computational complexity, and direct deployment on embedded platforms may introduce substantial latency. To address this issue, researchers commonly adopt lightweight encoders, low-resolution feature extraction, multi-scale feature reuse, knowledge distillation and boundary-aware losses to reduce computational cost while maintaining segmentation accuracy [45]. For navigation systems, not all pixel categories are equally important. In many scenarios, the system is more concerned with traversable areas, obstacle boundaries, dynamic objects and task-related targets than with complete and fine-grained semantic understanding of all categories. Therefore, navigation-oriented lightweight visual segmentation can adopt a task-driven strategy, in which high-accuracy modeling is focused only on categories closely related to motion safety and path planning, thereby reducing annotation cost and inference burden.

Vision alone provides incomplete geometry. Monocular depth is scale-ambiguous, stereo matching fails in weak or repetitive texture, and RGB-D sensing is range- and lighting-limited [46]. These weaknesses motivate complementary depth or motion sensing for stable spatial understanding.

Dynamic objects also violate the static-scene assumptions used by visual odometry and SLAM. Optical flow, tracking, temporal modeling, and motion-consistency tests can identify moving regions for risk prediction or exclusion from localization and mapping [47,48,49], but these functions must remain computationally affordable.

Third, vision-based perception also faces cross-scene generalization challenges. Many visual models perform well on public datasets, but their performance may degrade during real-world deployment due to changes in illumination, camera parameters, viewpoints, weather conditions, motion blur and scene category distributions [50,51]. For resource-constrained autonomous systems, frequent retraining or large-scale online fine-tuning are usually impractical. Therefore, stronger self-supervised learning, domain adaptation, few-shot adaptation and uncertainty estimation capabilities are required. Especially in long-term UAV and mobile robot operation scenarios, the perception system needs to maintain reliable outputs in unknown environments, rather than merely achieving high offline accuracy on fixed datasets.

Overall, vision-based task and scene perception provide resource-constrained autonomous systems with low-cost, information-rich environmental understanding, making it one of the most important perception foundations for intelligent navigation systems. Object detection, semantic segmentation, instance segmentation, traversable-area recognition and dynamic object perception support the autonomous navigation loop from the perspectives of objects, regions, instances and risks, respectively. However, relying solely on vision remains insufficient to fully address challenges related to three-dimensional geometry, scale recovery, occlusion handling, and robustness in complex dynamic environments.

### 3.3. 2D–3D Joint Perception and Semantic Understanding

As discussed in the previous section, vision-based perception provides rich semantic information for resource-constrained autonomous systems at relatively low cost. However, two-dimensional images alone cannot fully meet the spatial reasoning requirements of autonomous navigation. Images describe texture, color, and category-level semantics effectively, but do not directly provide stable depth, scale or spatial occupancy information. Navigation systems must therefore determine not only whether an obstacle or target is present, but also its three-dimensional position, size, orientation, distance and relationship with traversable space [52,53,54]. This requirement motivates the transition from purely image-based perception to 2D–3D joint perception [55,56].

The basic principle of 2D–3D joint perception is to combine the semantic richness of images with the geometric information provided by depth maps, point clouds, voxels or spatial grids [57,58,59,60]. Image features support category recognition and boundary discrimination, whereas three-dimensional observations provide distance, scale, surface geometry, and occupancy relationships. Their combination enables the system to infer not only what is present in the scene, but also where it is located and whether it constrains navigation. Thus, 2D–3D joint perception serves as an important bridge between image understanding and navigation-oriented spatial reasoning.

The geometric relationship between a 3D point and its corresponding 2D image projection is usually described by the pinhole camera model:

(1) $$s\tilde{p}\;=\;K\;[R\;t]{\tilde{P}}_{w}\;,\;\tilde{p}\;=\;{\left[u,v,1\right]}^{T},{\tilde{P}}_{w}\;=\;{\left[X,Y,Z,1\right]}^{T}$$

where $\tilde{p}$ = [u, v, 1]ᵀ is the homogeneous image coordinate, ${\tilde{P}}_{w}$ = [X, Y, Z, 1]ᵀ is the homogeneous world coordinate, K ∈ ℝ^3×3^ is the camera intrinsic matrix, R ∈ SO(3) and t ∈ ℝ^3^ define the world-to-camera transformation and s > 0 is the projective scale.

Conversely, when the depth value (u, v) of a pixel is available, the corresponding 3D point in the camera coordinate system can be recovered by back-projection:

(2) $$P_{c}=d(u,v)K^{-1}\tilde{p},\tilde{p}={\left[u,v,1\right]}^{T}$$

where *P_c_* ∈ ℝ^3^ is the recovered 3D point in the camera coordinate system, d(u, v) denotes the depth at pixel (u, v), and $\tilde{p}={\left[u,v,1\right]}^{T}$ is the corresponding homogeneous pixel coordinate. This back-projection provides the geometric basis for RGB-D perception, multi-view feature projection, and 2D–3D semantic fusion.

**Figure 3:**
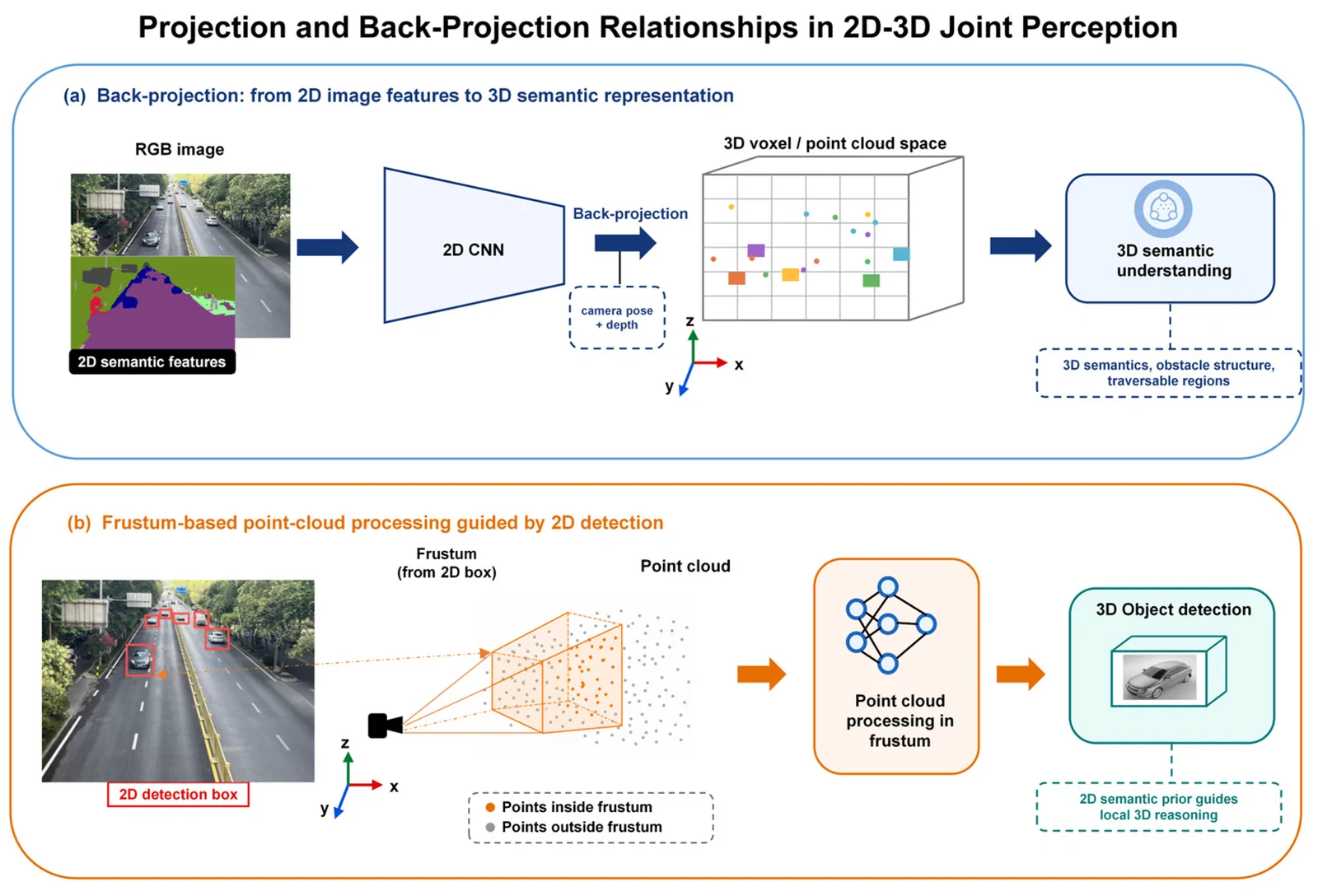
Projection and back-projection in 2D–3D joint perception: (**a**) image features are projected into voxel or point-cloud space for 3D semantic understanding; and (**b**) a 2D detection box defines a 3D frustum for local point-cloud processing and object estimation.

As illustrated in Figure 3, 2D–3D joint perception can be implemented through two representative paradigms: back-projecting image features into 3D space and using 2D detection priors to guide frustum-based point-cloud processing.

Multi-view methods such as 3DMV extract semantic image features and back-project them into voxel or point-cloud space using poses and depth [61]. Fusing these features with 3D geometry improves indoor semantic understanding without relying on raw RGB values alone.

Frustum PointNets represents a second strategy: a 2D detector defines a 3D frustum, and point-cloud segmentation and box estimation are restricted to that region [62,63,64]. This focuses computation on likely objects and is therefore attractive for resource-limited platforms.

A related approach projects pixel-level semantic scores or image features onto 3D points [65]. The resulting semantic point cloud combines image-based category cues with stable metric scale and geometry for detection, mapping, and obstacle recognition.

For navigation, the main benefit is a spatial semantic map that identifies both occupancy and object identity. Such maps support dynamic-object filtering, semantic planning, human–robot interaction, and long-term updating more directly than isolated image detections.

The main limitations are calibration and synchronization sensitivity [66,67,68], the cost of back-projection and 3D processing, inconsistency caused by moving objects, and mismatched sensor fields of view or resolution. These factors can produce incorrect semantic placement or uncertain coverage.

Practical systems should therefore use sparse or region-focused 3D processing, lightweight point-cloud networks, local maps, keyframes, online calibration, and uncertainty modeling to balance spatial consistency with onboard cost.

### 3.4. Camera-LiDAR Fusion for Three-Dimensional Scene Understanding

Among various 2D–3D joint perception schemes, Camera-LiDAR fusion is one of the most representative technical routes for three-dimensional environmental understanding in current autonomous systems [69,70,71]. Cameras provide high-resolution texture, color and semantic information, making them suitable for object category recognition, semantic segmentation and fine-grained appearance understanding. LiDAR, in contrast, directly measures three-dimensional distance and provides accurate spatial geometry, obstacle boundaries and scale information. The complementarity between the two modalities is evident: cameras “understand” the scene but provide unstable depth, whereas LiDAR “measures” the scene accurately but has limited semantic representation capability. Therefore, combining image semantics with LiDAR geometry can significantly improve the environmental understanding capability of autonomous systems in three-dimensional object detection, obstacle perception, free-space recognition, and semantic map construction.

Compared with the general 2D–3D joint perception discussed in the previous section, Camera-LiDAR fusion places greater emphasis on sensor-level complementarity and engineering deployment on real autonomous platforms. The fused three-dimensional scene representation can not only localize obstacles, but also infer their categories, spatial dimensions, motion risks and associated traversable regions. This makes Camera-LiDAR fusion particularly valuable in complex outdoor environments, semi-structured industrial environments, and urban traffic scenarios.

In terms of fusion levels, Camera-LiDAR fusion methods can generally be divided into data-level fusion, feature-level fusion, decision-level fusion and unified spatial representation fusion. Data-level fusion mainly projects LiDAR point clouds onto the image plane through extrinsic calibration or assigns image colors and semantic labels to three-dimensional points to form image-enhanced point clouds. For Camera-LiDAR fusion, a LiDAR point is first transformed into the camera coordinate system using the extrinsic calibration parameters:

*P_c_* = *R_cl_P_l_* + *t_cl_* (3)

where *P_l_* and P_c_ denote the same 3D point in the LiDAR and camera coordinate systems, respectively and R_cl_ and t_cl_ denote the rotation and translation from the LiDAR frame to the camera frame. Combined with Equation (1), this transformation enables LiDAR points to be projected onto the image plane for point coloring, semantic point-cloud generation and cross-modal feature alignment.

Data-level fusion is intuitive but calibration-sensitive. Feature-level methods align learned image and point-cloud features and offer stronger semantic–geometric modeling at higher computational cost. Decision-level methods combine independently produced boxes or confidence scores, improving modularity but losing some low-level complementarity. Unified BEV fusion transforms both modalities into a shared planning-oriented coordinate system.

Modern feature-level systems use projection, attention, or Transformer interactions to combine image semantics with 3D structure [72,73,74]. Their central difficulty is preserving LiDAR geometry while aligning features across different resolutions and coordinate systems; direct image-to-point or voxel-to-image projection can amplify sparsity, calibration error, or information loss [75].

BEV fusion aligns multimodal features in a top-down space suited to detection, mapping, and planning. BEVFusion [76] demonstrates this shared representation for multiple perception tasks [77,78]. Resource-limited platforms can reduce cost through local, sparse, or lower-resolution BEV grids focused on navigation-relevant regions.

Beyond detection, Camera–LiDAR fusion supports free-space recognition and semantic mapping: LiDAR constrains distance and obstacle geometry, while images distinguish roads, vegetation, water, walls, and dynamic objects.

Deployment remains limited by LiDAR cost, payload, power, sparse measurements, and field of view. Fusion also requires stable extrinsic calibration and synchronization [79,80,81], while voxelization, 3D convolution, attention, and BEV transformation place substantial demands on embedded memory and compute.

Overall, Camera-LiDAR fusion provides autonomous systems with a three-dimensional scene perception solution that combines semantic understanding and geometric measurement. It is an important technical pathway from two-dimensional visual perception toward three-dimensional navigation-oriented perception. Its main advantage lies in leveraging the semantic representation capability of cameras and the spatial ranging capability of LiDAR to achieve more reliable three-dimensional object detection, obstacle perception, free-space recognition and semantic map construction. However, due to limitations in sensor cost, calibration and synchronization, computational overhead and modality degradation, its deployment on small resource-constrained platforms still requires further lightweight and robust design.

### 3.5. Unified Environmental Representation Based on BEV and Occupancy

As autonomous systems move from isolated perception tasks toward continuous navigation in complex environments, two-dimensional bounding boxes, semantic segmentation masks, or local three-dimensional object boxes alone are no longer sufficient to meet the requirements of planning and obstacle avoidance. What navigation systems truly require is a unified, stable, updatable and planning-friendly spatial representation of the surrounding environment. Driven by this requirement, bird’s-eye-view (BEV) representation and three-dimensional occupancy representation have gradually become important directions in the evolution of intelligent perception toward the navigation closed loop [82]. Unlike conventional image-plane perception, BEV and occupancy representations no longer focus only on “what objects are present in the image”, but further describe “how the surrounding space is organized”, “which regions are occupied”, “which regions are traversable”, and “how dynamic objects may evolve”.

The core idea of BEV representation is to map multi-view images, LiDAR point clouds or multimodal features into a unified top-down coordinate system, thereby forming a spatial representation that is naturally consistent with ground motion, path planning and local obstacle avoidance. For ground mobile robots, AGVs and intelligent vehicles, BEV can intuitively represent road boundaries, obstacle locations, lane structures and traversable regions of dynamic objects. For low-altitude UAVs, local BEV representations can also be used to describe nearby obstacles, flight corridors and safe passage regions. Compared with image-plane detection, BEV representation reduces the scale variation caused by perspective projection and enables multi-view and multi-sensor information to be fused in a unified spatial domain. Therefore, it is more suitable for interfacing with planning modules.

(4) $$i=\left\lfloor \frac{x-x_{\min}}{\Delta x}\right\rfloor ,j=\left\lfloor \frac{y-y_{\min}}{\Delta y}\right\rfloor$$

where (i, j) denotes the integer BEV grid index; *x_min_* and *y_min_* are the lower bounds of the region of interest; Δx and Δy are the grid resolutions along the two horizontal axes.

Learned BEV systems transform multi-view images through depth estimation, geometry, or attention, while multimodal and cooperative variants add LiDAR, radar, or shared information [83,84,85,86]. BEVFormer [87] and BEVFusion support detection, segmentation, motion prediction, and related tasks in a shared space [88]. Their multi-view transformation and temporal fusion costs motivate lightweight backbones and sparse local modeling.

**Figure 4:**
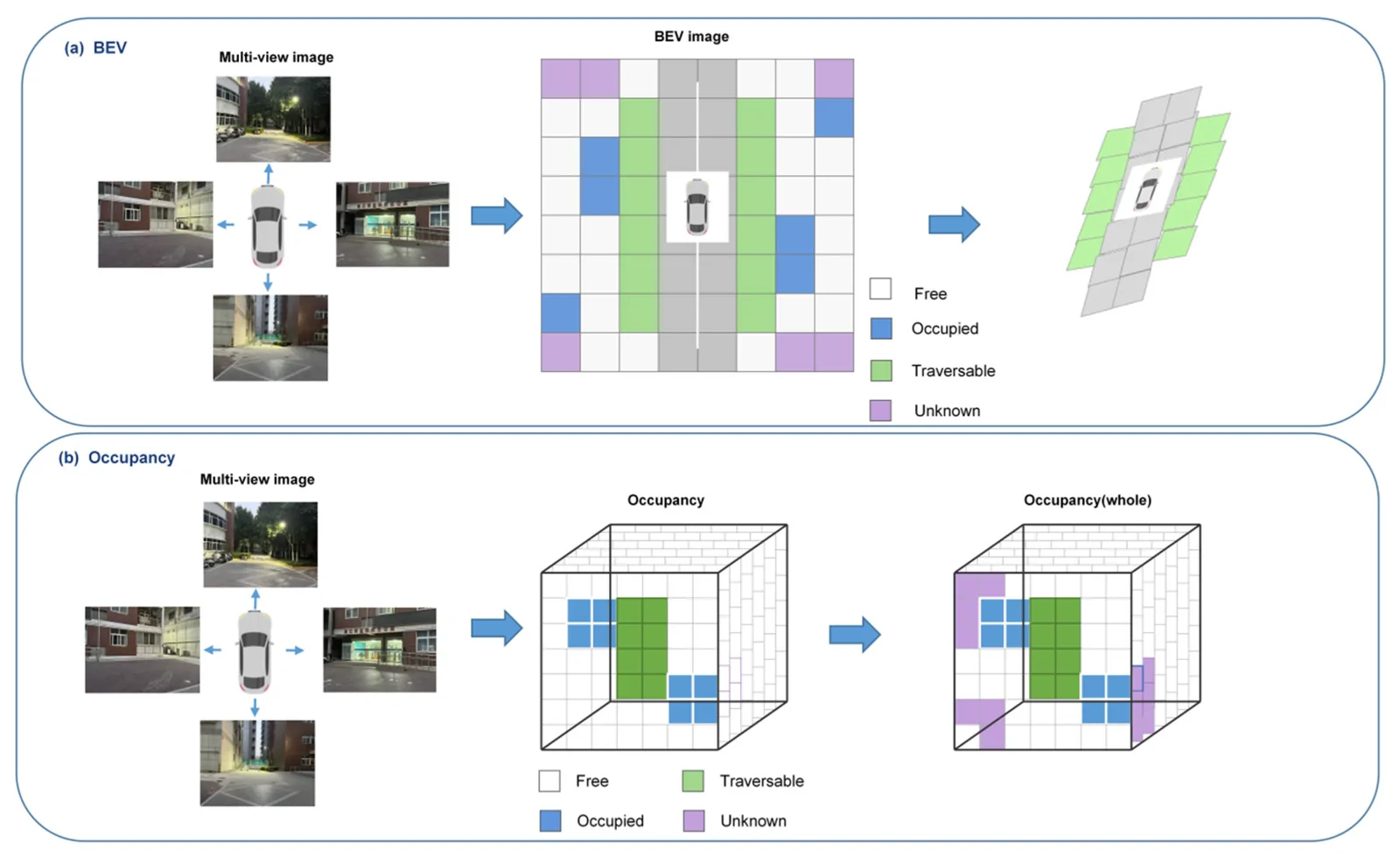
Comparison of BEV and 3D occupancy representations: (**a**) BEV compresses vertical structure into a top-down grid; and (**b**) occupancy preserves the 3D free, occupied, unknown, and traversable states, the colored cells denote different grid or voxel states: white indicates free space, blue indicates occupied space, green indicates traversable space, and purple indicates unknown or unobserved space. The gray cells show the reference road or ground grid rather than an additional occupancy class.

Occupancy extends BEV into 3D by predicting free, occupied, unknown, and optionally semantic states, as shown in Figure 4. It preserves vertical structures such as branches, wires, stairs, and overhangs and therefore supports UAV and indoor navigation more completely than object boxes alone.

Occupancy representation can be formulated as probabilistic estimation over discretized 3D space [89,90]:

*O*(*x*) = *P*(*m_x_* = 1|*z*_1:*t*_), *m_x_* ∈ {0,1} (5)

where O(x) denotes the occupancy probability at spatial location or voxel x; *m_x_* ∈ {0, 1} is the binary occupancy variable, with *m_x_* = 1 for occupied and *m_x_* = 0 for free; and *z*_1:*t*_ denotes the sensor observations accumulated from the initial time to time t.

BEV and occupancy remain memory- and compute-intensive, and vision-based variants depend on depth, pose, and calibration quality. Dynamic objects further complicate temporal updates. Local BEV, sparse or hierarchical occupancy, region-focused resolution, and selective edge–cloud updating can reduce these costs while retaining navigation-relevant structure.

### 3.6. Summary

Intelligent perception is progressing from image-level recognition toward spatial representations that can be used directly by planning. For resource-constrained platforms, the central design problem is to retain the semantic and geometric information needed for navigation while controlling calibration sensitivity, computation, memory, and energy use.

## 4. Learning-Enhanced State Estimation

### 4.1. State Estimation Challenges in Autonomous Navigation

In the closed-loop of autonomous navigation, intelligent perception primarily addresses the question, “What exists in the external environment?” In contrast, state estimation answers, “Where is the autonomous system, how is it moving, and how reliable are the estimates?” For UAVs, mobile robots, AGVs, and other resource-constrained autonomous platforms, state estimation typically requires continuous outputs of position, orientation, velocity, angular velocity, sensor biases and associated uncertainties, providing real-time feedback for mapping, obstacle avoidance, path planning and motion control [91,92]. While perception provides constraints from the external environment, state estimation conveys the spatiotemporal relationship between the system and its surroundings. Hence, state estimation forms a critical link between perception and motion decision-making.

Unlike conventional offline perception tasks, state estimation in autonomous navigation is continuous, real-time, and closed-loop. The system must not only estimate poses at individual time instances but also maintain temporally consistent trajectories and limit drift during extended operation. Indoor mobile robots must perform local positioning without GNSS, relying on sensors such as cameras, LiDAR, IMUs, wheel odometry or UWB. UAVs require high-frequency state feedback under conditions of rapid motion, vibration, swift attitude changes and limited payload. Outdoor mobile platforms must handle GNSS occlusions, multipath effects, visual degradation, sparse LiDAR data and IMU drift. Thus, the challenge of state estimation extends beyond accuracy to robustness, real-time performance and long-term stability.

Mathematically, state estimation for autonomous systems can be formulated as a dynamic system inference problem under uncertainty. The system predicts the next state using a motion model and corrects it based on sensor observations. Motion models typically incorporate system dynamics, odometry, IMU preintegration or control inputs, while observation models derive from cameras, LiDAR, GNSS, UWB or environmental feature matching. Under ideal conditions—accurate system models, known noise statistics and reliable observations—classical filtering and optimization methods can achieve satisfactory estimates. However, in real-world navigation scenarios, noise is often non-stationary, observations may intermittently fail, and motion models may deviate due to terrain, vibration, payload variations and actuator errors, making the state estimation problem significantly more complex than standard filtering formulations [93].

Resource-constrained autonomous systems impose additional engineering requirements on state estimation. Low-cost platforms often cannot accommodate high-precision, high-power sensors, relying instead on compact IMUs, lightweight cameras and small LiDAR, resulting in limited observation quality and pronounced noise variability. Embedded computing platforms may not support prolonged execution of complex global optimization or dense mapping algorithms, necessitating high-frequency state estimation within limited computation, memory and power budgets. For high-speed aerial or ground platforms, latency in state estimation directly impacts control stability and safety. Therefore, state estimation for resource-constrained systems is not solely about maximizing accuracy but achieving a balance among precision, robustness, real-time performance and computational efficiency.

Traditional state estimation methods form the foundation of modern autonomous navigation, including Kalman filters and their nonlinear extensions, particle filters, visual odometry, visual-inertial odometry, LiDAR odometry and factor graph-based optimization techniques [94,95]. While these methods offer strong theoretical foundations and engineering interpretability, their performance deteriorates as autonomous systems transition from structured environments to dynamic, open, complex, resource-limited scenarios. In particular, conventional methods exhibit limitations in noise adaptation, nonlinear modeling, handling sensor degradation and cross-domain generalization. Learning-enhanced state estimation has emerged to address these challenges by retaining the interpretability and stability of classical estimators while leveraging data-driven models to adaptively capture noise characteristics, nonlinear dynamics, observation reliability, and environmental variations.

**Figure 5:**
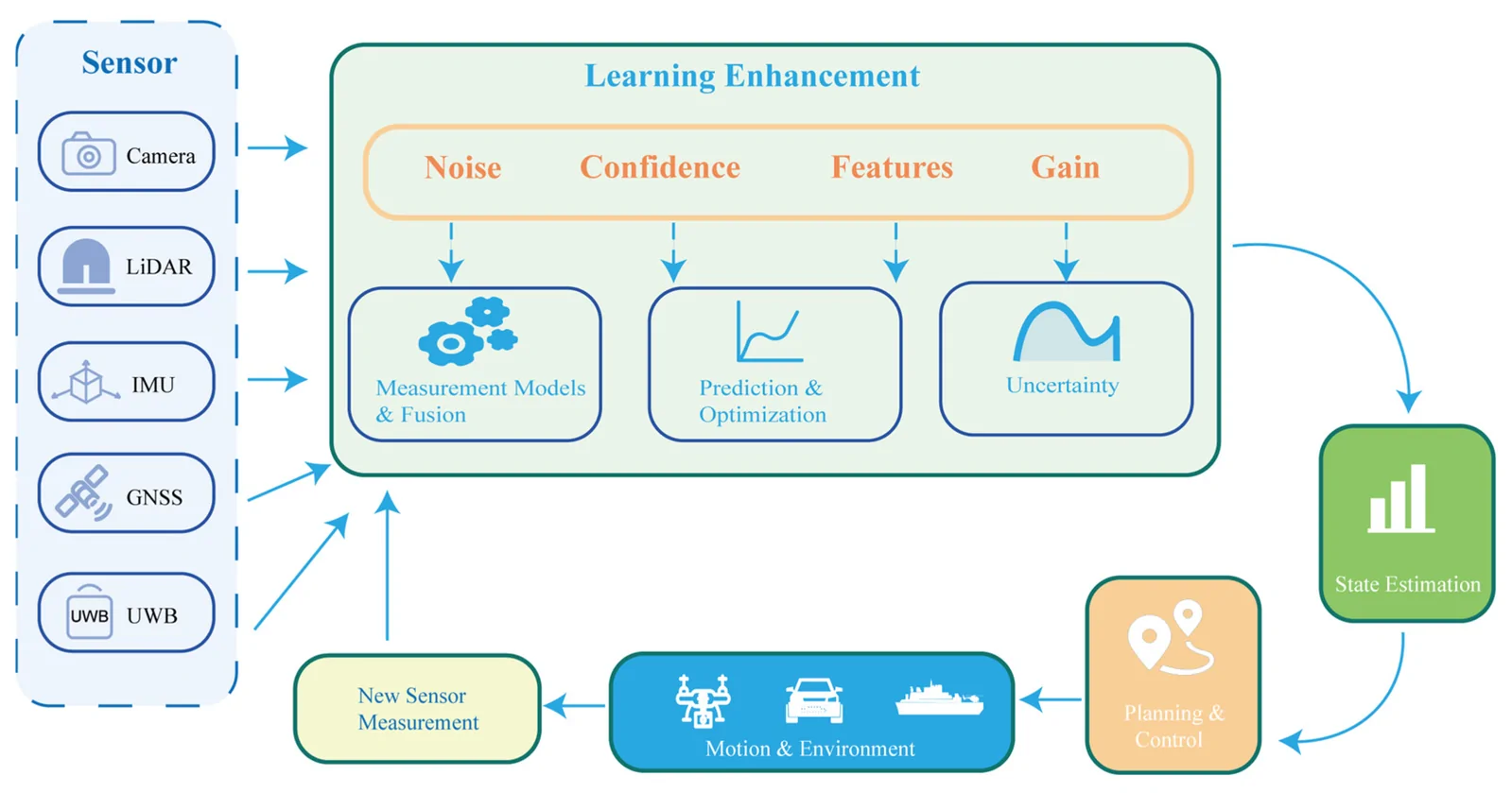
Learning-enhanced state estimation with multimodal fusion embedded within the estimator and closed-loop feedback through planning, control, platform motion, and new sensor measurements.

As shown in Figure 5, multimodal observations are fused within the state estimator, while learning-based modules enhance measurement modeling, prediction and optimization, and uncertainty estimation. The resulting state estimate supports planning and control within a closed feedback loop through platform motion and new sensor measurements.

### 4.2. Limitations of Conventional Filtering and Optimization Methods

Classical estimators include Kalman-filter variants, particle filters, and nonlinear VIO/SLAM back ends [96,97]. They provide recursive or optimization-based inference but depend on model fidelity, noise assumptions, initialization, and affordable computation.

Fixed covariance models are often inadequate because IMU noise changes with temperature and vibration, visual quality changes with texture and illumination, GNSS suffers blockage and multipath, and LiDAR degrades in weather or geometric degeneracy. Incorrect weighting can produce inconsistent estimates, drift, or divergence.

Nonlinearity creates additional error. EKF linearization may become inconsistent under rapid motion or poor initialization; UKF complexity grows with state dimension; and particle filters trade posterior fidelity against particle degeneracy and computational cost. These limitations are acute on embedded platforms.

VIO and SLAM also depend on stable tracking, correct association, IMU preintegration, and bounded optimization [98,99]. Low texture, illumination change, dynamics, and bias errors can corrupt constraints, while global optimization is expensive and small sliding windows sacrifice long-term information.

Furthermore, conventional state estimation methods often lack the ability to actively evaluate observation quality and scene changes. Many methods assume that sensor measurements follow predefined noise models. In practical operation, however, local sensor failure, outlier measurements, dynamic interference and cross-domain distribution shifts frequently occur. If the system cannot determine whether visual features are reliable, whether GNSS measurements are contaminated by multipath effects or whether LiDAR matching is degraded at a given moment, erroneous observations may be introduced into the state update process, resulting in biased estimates or even system failure. For safety-critical autonomous systems, outputting state estimates alone is insufficient; it is also necessary to assess estimation uncertainty, observation reliability and potential failure risks.

Therefore, learning-enhanced state estimation is not intended to completely replace conventional filtering and optimization frameworks. Instead, it aims to compensate for their limitations in noise modeling, nonlinear representation, observation quality assessment and rapid adaptation by introducing data-driven models. A more reasonable direction is to combine the physical constraints, probabilistic inference and interpretable structures of classical state estimation with the feature extraction, adaptive modeling and nonlinear approximation capabilities of deep learning. Following this idea, recent studies have explored adaptive noise learning, neural Kalman filtering, differentiable particle filtering, learning-assisted VIO/SLAM and uncertainty estimation.

### 4.3. Adaptive Filtering and Noise Learning

As discussed in Section 4.2, fixed process and measurement noise models can become unreliable when motion, environment, or sensor quality changes [100,101]. Adaptive filtering therefore estimates covariance or observation weights online from residuals and sensor-quality cues rather than relying solely on fixed parameters.

Adaptive filters update covariance or observation weights from innovations, residuals, or sensor-quality cues. Rising residuals may increase measurement covariance, while model mismatch may increase process covariance. These interpretable rules are inexpensive but often manually tuned and may not generalize to nonlinear, tightly coupled systems.

Learned modules can instead estimate noise or confidence from image quality, feature tracking, IMU vibration, GNSS signal indicators, or LiDAR registration residuals. They preserve the filter structure while adapting measurement influence to current conditions.

Neuro-fuzzy models, adaptive networks, LSTM/GRU models, and compact CNNs or multilayer perceptrons can learn covariance, temporal noise, or quality weights [102,103]. Compared with end-to-end estimation, this hybrid design is easier to interpret and integrate onboard.

The main benefit is targeted adaptation with limited architectural change. Risks include domain dependence, physically implausible covariance, and incorrect confidence estimates. Predicted covariance must remain positive definite and calibrated, and learned updates should be checked against residuals, physical priors, and online consistency tests.

Overall, adaptive filtering and noise learning represent one of the most practically deployable categories of learning-enhanced state estimation. They retain the recursive structure and interpretability of conventional filters while using data-driven models to improve noise modeling and observation quality assessment. However, when the system faces stronger nonlinearities, more complex observation models or intrinsic limitations in the traditional filtering update structure, dynamically adjusting noise parameters alone becomes insufficient. In such cases, neural networks need to participate more directly in Kalman gain estimation, state updates, particle weighting and observation model learning, which motivates the development of neural Kalman filtering and differentiable particle filtering.

### 4.4. Neural Kalman Filtering and Differentiable Particle Filtering

Neural Kalman methods retain the prediction–update structure while learning difficult components such as gains, residual corrections, transition errors, or covariance mappings. KalmanNet, for example, combines recursive structure with data-driven nonlinear adaptation and is applicable to visual–inertial, GNSS/INS, and low-cost inertial estimation.

Neural Kalman filtering frameworks, represented by methods such as KalmanNet [104], usually do not completely discard the conventional filtering structure. Instead, they embed neural networks into the prediction or update stage [105], enabling the estimator to learn more appropriate state correction strategies from historical states, observation sequences and innovation information. This hybrid structure provides two major advantages. On the one hand, the Kalman filtering framework preserves the basic structure of recursive estimation and uncertainty propagation, making the method more interpretable than a purely end-to-end black-box network. On the other hand, neural networks can learn complex nonlinear relationships from data, thereby alleviating the performance degradation of conventional filters under inaccurate models, noise mismatch, and complex observation conditions. For tasks such as visual-inertial estimation, GNSS/INS fusion and low-cost IMU-based state estimation, neural Kalman filtering provides a flexible enhancement pathway for classical estimation frameworks.

Particle filters represent nonlinear, non-Gaussian, or multimodal posteriors but suffer from degeneracy and high sampling cost. Differentiable particle filters learn observation scores, proposal distributions, or particle weights through differentiable approximations, improving high-dimensional localization or relocalization while retaining particle-based uncertainty.

Differentiable particle filtering attempts to integrate the particle filtering process with neural network training mechanisms [106,107]. Its basic idea is to formulate particle propagation, observation scoring, weight updating and certain resampling operations as differentiable or approximately differentiable computational processes, so that neural networks can learn observation models, particle weight assignment or proposal distributions through backpropagation. In this manner, the system can use deep features to score and select particles more effectively, reducing invalid particles and improving sampling efficiency. For tasks such as visual global localization, robot relocalization and semantic map matching, differentiable particle filtering can combine the ability of particle filters to represent complex posterior distributions with the ability of deep networks to extract high-dimensional observation features.

Neural Kalman filters are generally better suited to compact, high-rate local estimation, whereas differentiable particle filters better represent global or multimodal uncertainty but require more computation and stable training. Both need lightweight models and bounded updates for embedded use.

Filter divergence and statistical inconsistency. Neural filters can outperform model-based baselines under the nonlinearities and model mismatch represented in their evaluation data, but a learned gain, covariance, likelihood, or particle weight does not by itself guarantee stable recursive error dynamics. Small biased updates may accumulate over long horizons, while overconfident uncertainty estimates can cause an inconsistent state estimate to appear reliable. Comparative experiments on differentiable filters show that training stability and the choice of uncertainty model, initialization, loss function, and hyperparameters materially affect accuracy and uncertainty estimates [108]. Evaluations should therefore report full-run divergence or failure rates, maximum and percentile errors, innovation statistics, confidence-interval coverage or covariance calibration and, where the probabilistic assumptions permit, normalized estimation error squared (NEES) and normalized innovation squared (NIS), rather than relying only on average RMSE.

Physical and probabilistic consistency. Learned state corrections may violate kinematic constraints, coordinate-frame conventions, rotation-manifold structure, or quaternion normalization, and unconstrained covariance outputs may fail to remain symmetric positive definite. These risks can be reduced by applying learning modules to bounded residual corrections or soft observation weights, retaining analytical prediction and update structure, enforcing state-manifold operations, parameterizing covariance through a Cholesky factor or another positive-definite construction, and checking learned updates against physical and innovation residuals. Experiments on multivariate learned uncertainty show that the form and calibration of predicted covariance can substantially affect filtering performance under both in-domain and out-of-domain data [109]. Consequently, a low trajectory error alone should not be interpreted as evidence of a physically or statistically consistent estimator.

Distribution shift in sensors, dynamics, weather, or failures can make an offline-trained estimator biased or overconfident [110]. Validation should include cross-dataset/device tests, altered calibration and noise, dropout, and environmental shifts, reporting failure frequency, tail error, calibration, and recovery time. Unreliable learned updates should be bounded or rejected in favor of a validated model-based fallback.

### 4.5. Learning-Assisted VIO and SLAM

Operational estimators are usually embedded in VO, VIO, LiDAR odometry, SLAM, or factor-graph systems that maintain features, keyframes, local maps, and temporal constraints [111,112]. Their geometric structure is interpretable, but tracking, association, and mapping can fail under weak texture, illumination change, dynamics, rapid motion, or sensor degradation.

Learning-assisted systems target these vulnerable components rather than replacing the entire estimator [113]. Learned keypoints, descriptors, matching, and optical flow can outperform handcrafted ORB [114], FAST [115], or Shi–Tomasi [116] features under difficult appearance changes, although embedded use still requires sparse selection and low-latency networks.

Learned depth can assist monocular scale recovery as a prior or soft constraint, but its scale and geometry may not transfer to unseen environments; geometric triangulation and multi-view consistency should therefore remain available.

Semantic or motion segmentation can remove dynamic features that would otherwise bias pose and contaminate maps. The same information can enrich maps with objects, traversability, and scene structure for longer-term planning.

**Figure 6:**
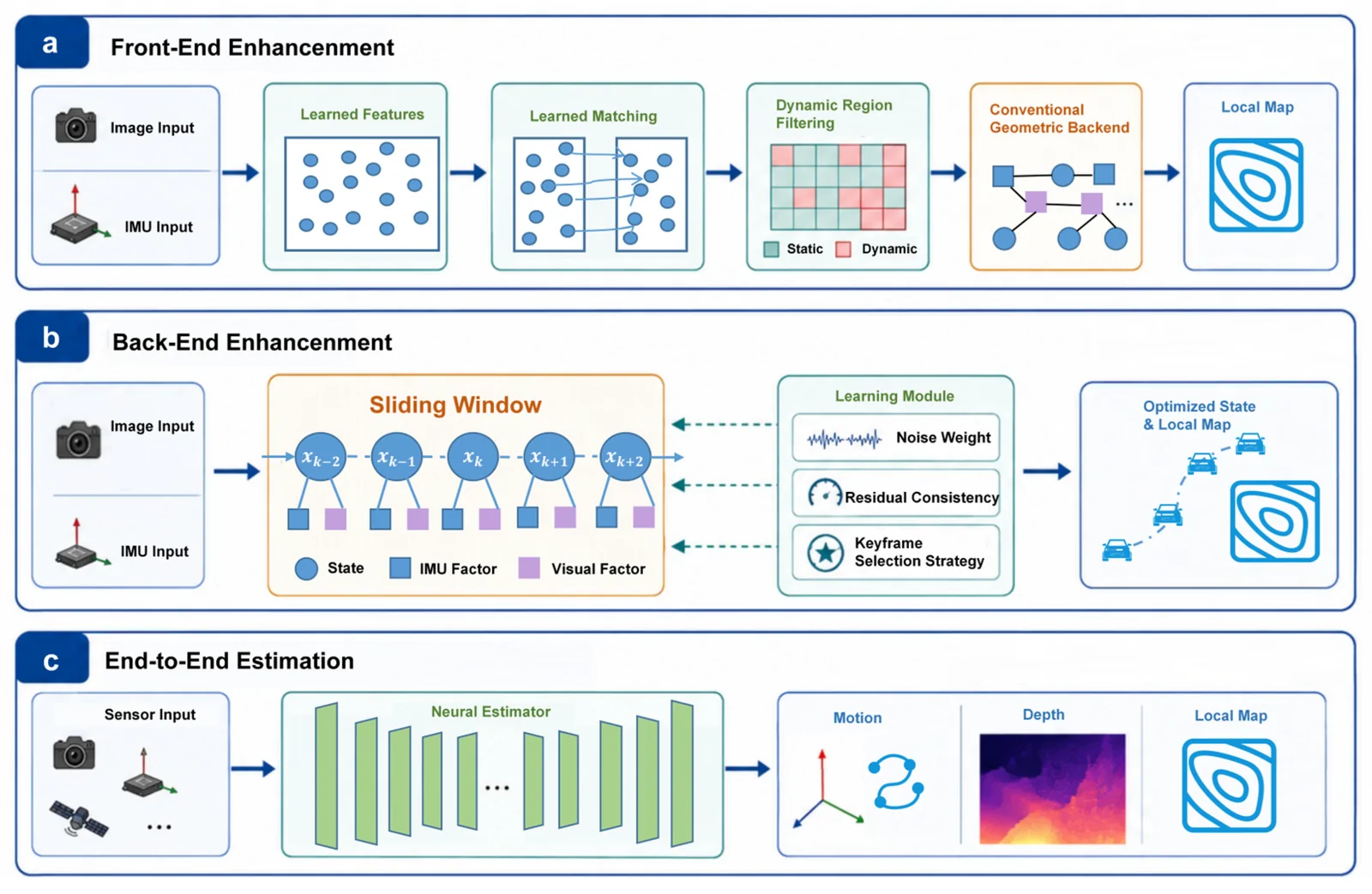
Learning-assisted VIO and SLAM architectures: (**a**) learned front-end features and dynamic-region filtering with a geometric back end; (**b**) learned weights, residual confidence, or keyframe selection within optimization; and (**c**) end-to-end motion, depth or local-map estimation.

Figure 6 distinguishes front-end enhancement, back-end assistance within geometric optimization, and end-to-end estimation. The first two preserve more explicit geometry and are generally easier to interpret and validate.

In addition, learning-based methods can enhance loop closure detection, relocalization and failure monitoring. Traditional loop closure detection often relies on bag-of-words models or handcrafted feature similarity, which may lead to missed detections or false matches under illumination changes, viewpoint variations and repetitive scene structures. Deep global descriptors and scene representation learning can improve the robustness of place recognition, thereby strengthening loop closure constraints and global consistency during long-term operation. For autonomous systems, learning modules can also estimate whether the current state estimation is reliable based on feature tracking quality, residual variations, map matching scores and sensor states, enabling localization failure warning and safety degradation.

From a system architecture perspective, learning-assisted VIO/SLAM can be broadly divided into three categories. The first category is front-end enhancement, where learning-based methods improve feature extraction, matching, depth estimation and dynamic region recognition, while the back end still relies on conventional geometric optimization. The second category is back-end assistance, where noise weights, residual confidence or keyframe selection strategies are learned to improve the robustness of sliding-window optimization and factor graph optimization. The third category is end-to-end or semi-end-to-end estimation, where neural networks directly predict camera motion, depth or local maps. However, the interpretability, generalization ability and safety verification of such methods remain challenging. For resource-constrained autonomous systems, the first and second categories are usually more practical from an engineering perspective, because they preserve the stability of traditional estimation frameworks while enhancing vulnerable components with relatively small learning modules.

Overall, learning-assisted VIO and SLAM represent an important transition of learning-enhanced state estimation from the filtering level to the system level. Their advantages lie in improving front-end feature robustness, enhancing adaptability to dynamic environments, strengthening loop closure and relocalization performance, and supporting semantic map construction. However, their limitations are also evident: learning modules increase computational burden, training data distributions affect generalization, the uncertainty of deep network outputs is difficult to rigorously integrate with geometric optimization, and end-to-end methods still lack sufficient interpretability for safety-critical scenarios. Therefore, for resource-constrained platforms, the future direction is not fully black-box neural SLAM, but rather a deeper coupling between lightweight learning modules and geometric optimization frameworks.

### 4.6. Summary

Learning-enhanced state estimation is most useful when compact learning modules improve noise adaptation, observation assessment, or vulnerable VIO/SLAM components while classical filters and geometric optimization retain physical structure. Its deployment value therefore depends on generalization, uncertainty handling, computational cost, long-horizon consistency, and a validated fallback path rather than offline accuracy alone.

## 5. Multimodal Navigation and Sensor Fusion Technologies

### 5.1. Fundamental Problems of Multimodal Navigation Fusion

Intelligent perception describes the external environment, whereas state estimation determines the platform pose, velocity, sensor biases, and uncertainty. In practical navigation systems, these functions are closely coupled. Cameras, IMUs, GNSS, LiDAR, radar, UWB, wheel odometers, event cameras, and other sensors provide complementary information for localization, mapping, obstacle avoidance, planning, and control [117,118,119].

Different sensors have different sampling rates, coordinate frames, latency, observability, noise characteristics, and failure modes [120]. Improper synchronization, calibration, data association, or uncertainty modeling may introduce inconsistent constraints and degrade the navigation solution. Multimodal fusion must therefore assess sensor reliability, reject abnormal observations, assign appropriate weights, and propagate uncertainty rather than simply combine sensor data.

**Figure 7:**
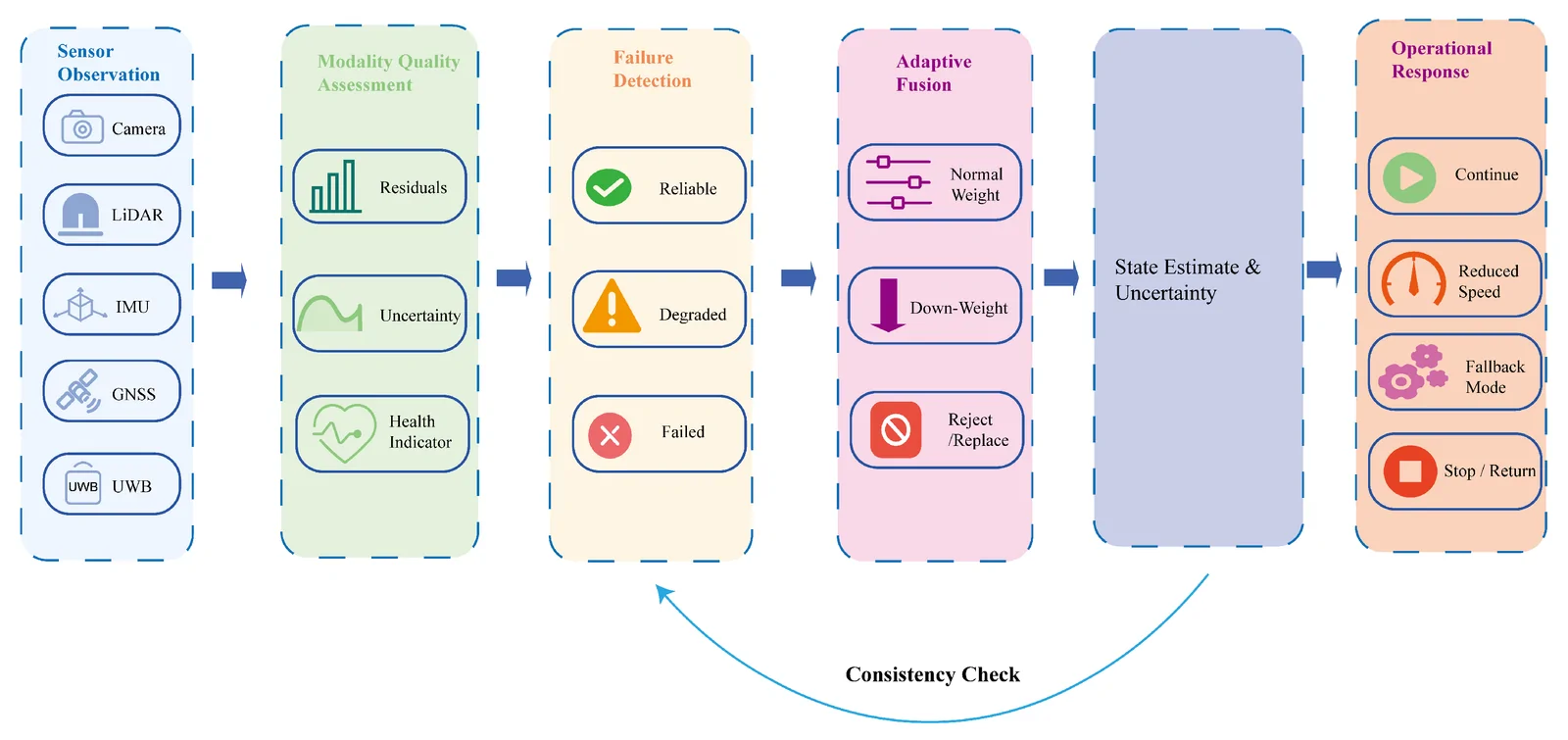
Failure-aware multimodal fusion linking sensor-quality assessment, failure detection, adaptive fusion, state uncertainty, consistency feedback, and operational response.

As shown in Figure 7, observations are evaluated using residuals, uncertainty, and health indicators and classified as reliable, degraded, or failed. Their contributions are normally weighted, down-weighted, or rejected. The resulting state and uncertainty inform normal operation, reduced speed, fallback, or safe stop, while consistency feedback updates these decisions as conditions change.

Fusion may occur at the data, feature, state, factor-graph, map, or decision level, while learning-based policies may support downstream navigation decisions. On resource-constrained platforms, fusion must also consider payload, power, bandwidth, computation, memory, and latency [121,122,123,124]. Therefore, sensing and computational resources should be allocated according to task relevance and current sensor health.

### 5.2. Typical Sensor Complementarity and Fusion Levels

Sensor roles are complementary. Cameras provide semantics and visual odometry but degrade with lighting, blur, occlusion, or weak texture. IMUs provide high-rate motion but drift; GNSS provides global position but fails under blockage or multipath; LiDAR supplies metric geometry at substantial compute and memory cost; radar remains robust in adverse visibility; and UWB provides infrastructure-dependent indoor ranging. Table 4 consolidates these complementary characteristics along four dimensions—the information each sensor provides, its strengths, its degradation modes, and its typical navigation use—and shows that no single modality remains reliable across all operating conditions.

The complementary characteristics of different sensors determine the basic forms of multimodal navigation fusion. Visual-inertial fusion combines visual spatial constraints with high-frequency IMU measurements and is widely used for local state estimation in UAVs and mobile robots. Visual-inertial-GNSS fusion introduces global position constraints to suppress long-term drift, making it suitable for outdoor long-range navigation. LiDAR-inertial fusion uses IMU measurements for motion compensation and short-term prediction, while LiDAR provides geometric matching and mapping constraints. LiDAR-visual-inertial fusion further integrates image semantics, point-cloud geometry and inertial motion information to improve robustness in complex three-dimensional environments. The suitability of these combinations depends on payload capacity, environmental structure, sensor cost, power consumption, computational resources and task requirements.

Fusion may occur at the data, feature, state, factor-graph, or map level. Earlier fusion retains richer information but demands calibration, synchronization, bandwidth, and computation. State and factor-graph fusion compactly combine pose constraints, while map-level fusion produces planning-oriented semantic, BEV, occupancy, or topological representations.

Resource-constrained systems should use hierarchical, task-oriented fusion: compact front ends extract critical observations, bounded filters or sliding windows combine state constraints, and only local or task-relevant maps are maintained.

### 5.3. Visual-Inertial and Visual-Inertial-GNSS Fusion

Visual–inertial fusion combines camera-based spatial constraints with high-rate IMU propagation [125]. Cameras drift or fail under poor appearance, whereas inertial estimates drift with bias; together they provide continuous, low-cost local pose estimation for UAVs and mobile robots.

Loosely coupled systems fuse completed visual-odometry outputs with IMU estimates, offering modularity but discarding raw constraints. Tightly coupled systems jointly estimate pose, velocity, biases, and features using reprojection and preintegration factors. VINS-Mono [126] and VINS-Fusion [127] use keyframes, marginalization, sliding windows, and loop or global constraints to balance robustness and real-time operation.

Despite its practical advantages, VIO mainly provides local relative localization. During long-term operation, accumulated errors may arise from visual mismatches, IMU bias drift, scale inconsistency, insufficient loop closure and environmental degeneracy. Monocular VIO is especially sensitive to scale observability and motion excitation, while stereo or RGB-D VIO can provide more direct depth constraints but may increase sensor cost, calibration complexity, and computational burden. In long-distance outdoor inspection, agricultural operation, logistics transportation and UAV route-following tasks, relying solely on VIO is often insufficient to maintain global trajectory consistency. Therefore, GNSS is commonly introduced into the visual-inertial framework to provide global absolute position constraints and suppress long-term drift.

Visual-inertial-GNSS fusion combines the high-frequency local continuity of VIO with the global drift-free property of GNSS positioning. In open-sky outdoor environments, GNSS can provide absolute position measurements that correct accumulated drift from visual-inertial odometry. Meanwhile, VIO can maintain smooth short-term motion estimation when GNSS updates are sparse or temporarily unavailable. This complementary relationship makes visual-inertial-GNSS fusion particularly valuable for outdoor UAVs, agricultural robots, inspection vehicles, autonomous delivery platforms, and other systems that require both local motion continuity and global positioning consistency.

GNSS may be fused as a position solution or as raw pseudorange, Doppler, or carrier-phase observations. Loose coupling is simpler but inherits GNSS solution errors; tight coupling uses partial satellite information more effectively but requires clock, frame, timing, outlier, and computational management.

Practical systems must synchronize high-rate VIO with lower-rate GNSS and reject blockage, multipath, and non-line-of-sight measurements through robust models, adaptive weighting, and innovation tests.

### 5.4. LiDAR-Inertial and LiDAR-Visual-Inertial Fusion

LiDAR–inertial fusion uses IMU propagation and motion compensation with scan registration, providing geometry-based estimation when vision is degraded by darkness, lighting, or weak texture [128,129].

IMU deskewing compensates motion during each LiDAR scan, after which point-to-line/plane or local-map registration corrects inertial drift. FAST-LIO [130] and LIO-SAM [131] represent filtering-based and smoothing-based designs. LIO is robust to visual degradation but becomes underconstrained in corridors, open fields, tunnels, or other geometrically degenerate scenes.

Adding vision supplies texture, semantics, and loop closure; LiDAR supplies metric geometry; and the IMU bridges their lower-rate updates [132,133]. This redundancy is useful when illumination, texture, or geometric structure selectively degrades one modality.

Unified factor graphs can combine reprojection, preintegration, scan matching, loop closure, GNSS, or wheel-odometry factors. LVI-SAM [134] illustrates this approach, which improves flexibility but increases calibration, association, synchronization, outlier-rejection, and scheduling demands.

LiDAR-related fusion methods face clear engineering costs on resource-constrained platforms. High-channel LiDAR sensors are often expensive, power-hungry and relatively heavy, which limits their use on small UAVs, microrobots and low-cost mobile platforms. Although low-channel LiDAR, solid-state LiDAR, and miniature depth sensors are becoming more accessible, they usually provide sparser point clouds, narrower fields of view or shorter sensing ranges. In addition, point-cloud preprocessing, voxel map maintenance, nearest-neighbor search, scan matching and factor graph optimization can impose substantial computational and memory burdens. These burdens are particularly significant for embedded platforms with limited GPU capability, memory capacity, and power budget.

Therefore, the practical deployment of LiDAR-inertial and LiDAR-visual-inertial fusion on resource-constrained systems requires lightweight design. Possible strategies include using low-channel or solid-state LiDAR sensors, adopting sparse point-cloud processing, maintaining only local submaps, reducing voxel resolution in noncritical regions, selecting keyframes adaptively and simplifying the back-end optimization problem. For small platforms, it is also necessary to decide whether the visual branch should be used for full dense semantic perception, lightweight feature tracking, loop closure or only failure recovery. A task-driven fusion design is usually more practical than a fully redundant multimodal architecture.

Overall, LiDAR-inertial and LiDAR-visual-inertial fusion can significantly improve geometric robustness and mapping capability in complex three-dimensional environments. LiDAR-inertial fusion is particularly suitable for visually degraded scenes and geometry-based localization, while LiDAR-visual-inertial fusion further enhances semantic understanding, loop closure and cross-modal robustness. However, their deployment value depends strongly on payload capacity, power budget, computational resources, sensor cost, and environmental structure. For resource-constrained autonomous systems, future development should emphasize lightweight LiDAR odometry, sparse geometric representation, adaptive modality weighting, online calibration and efficient integration of visual semantics with LiDAR-inertial back ends.

### 5.5. Modality Degradation, Failure Detection, and Resource-Constrained Challenges

Multimodal fusion does not necessarily mean that more sensors always lead to better navigation performance. In real autonomous systems, different sensors may degrade under different environmental and operational conditions. A robust fusion system must determine which modality should be trusted, which observation should be down-weighted, and how the system should operate when severe sensor failure occurs. Therefore, robust multimodal navigation fusion should not simply aggregate all available observations, but should selectively exploit reliable information according to sensor conditions and task requirements. Table 5 organizes the resulting strategies by failure mode and by the level at which they act—degradation indicators, estimator responses, and operational fallbacks—and records, for each, the supporting evidence and its remaining limitations. These three levels are discussed in turn below.

Detection and uncertainty assessment. Existing methods infer degradation from sensor-specific and cross-modal evidence rather than from sensor availability alone. Visual indicators include tracked-feature count and distribution, reprojection residuals, and estimator uncertainty; GNSS checks include satellite health, elevation, lock continuity, measurement residuals, and geometry; LiDAR checks use registration residuals and geometric observability; and inertial checks use saturation flags, bias or covariance growth, and disagreement with exteroceptive motion. VINS-Mono includes active failure detection and recovery, while GVINS screens unreliable satellite observations and handles GNSS loss and reacquisition within tightly coupled estimation. Learned confidence models may complement these checks, but their outputs should be verified against physical constraints and residual consistency before they affect safety-critical decisions [135].

**Table 5:** Sensor-degradation indicators, estimator responses, and operational fallbacks.

Failure Mode	Indicators	Estimator Response	Operational Response	Evidence and Limitation
Vision: low texture, lighting change, blur, occlusion, or feature loss	Feature count/distribution; reprojection or flow residuals; covariance growth	Reject/down-weight visual factors; propagate with other sensors; reinitialize or relocalize	Continue while uncertainty is bounded; otherwise slow, return, request intervention, or stop	VINS-Mono supports failure detection, recovery, and relocalization; prolonged loss still requires another observable modality or a safe response.
GNSS: blockage, multipath, NLOS, intermittent lock, or reacquisition	Satellite health/geometry; lock history; pseudorange/Doppler residuals; cross-modal innovation	Reject unstable satellites; robustly weight observations; use local VIO/LIO; restore GNSS after consistency checks	Use a monitored local frame temporarily; restrict missions requiring global integrity	GVINS addresses intermittent GNSS and reacquisition [136]; InGVIO uses tightly coupled GNSS–visual–inertial estimation [137]. Integrity bounds remain underreported.
LiDAR: degeneracy, sparse returns, registration failure, or limited field of view	Registration residuals; correspondence count; geometric observability; motion disagreement	Reduce/suspend LiDAR updates; propagate inertially; add visual, GNSS, or loop-closure constraints	Reduce speed or stop before uncertainty exceeds task-specific limits	FAST-LIO and LIO-SAM add inertial constraints; LVI-SAM and Super Odometry add vision. Validated fallback thresholds remain limited.
IMU, calibration, or timing faults	Status flags; bias/covariance growth; preintegration residuals; cross-modal disagreement	Reject corrupted intervals; re-estimate bias, timing, or extrinsics; use independent observations	Enter a validated low-dynamics mode or safe stop if inertial propagation remains unreliable	IMU calibration is established [138,139], and VINS-Mono includes recovery; complete IMU failure and closed-loop recovery are rarely evaluated.

Note: The cited studies demonstrate estimator-level detection or recovery; operational fallbacks remain platform-dependent and are seldom validated end-to-end.

Estimator-level mitigation. Once a measurement is judged unreliable, an estimator can reject the observation, increase its covariance, apply a robust loss, disable the corresponding factor, reinitialize affected states, or rely temporarily on complementary modalities. Tightly coupled VIO and LIO systems illustrate how inertial propagation bridges short gaps in visual or LiDAR updates, while LVI-SAM and Super Odometry introduce additional visual and LiDAR constraints to reduce dependence on a single exteroceptive modality. Such redundancy can improve continuity, but it does not by itself establish a safety guarantee because the remaining state may become weakly observable or correlated failures may affect several modalities simultaneously.

System-level degradation and emergency response. Estimator uncertainty and modality-health flags should be propagated to planning and control. Depending on the remaining observability, a platform may continue in a bounded local mode, reduce speed, enlarge safety margins, return home, request intervention, or stop. These actions should be linked to explicit thresholds, hysteresis, and recovery conditions. The reviewed literature provides evidence for estimator recovery, relocalization, and continued estimation during some single-modality degradations, but controlled comparisons of complete sensor loss, simultaneous failures, false alarms, recovery time, and closed-loop emergency behavior remain limited. These actions therefore remain engineering recommendations and open validation requirements rather than established safety guarantees.

Resource-constrained platforms further amplify the engineering difficulty of multimodal fusion. Multi-sensor systems increase hardware cost, weight, volume, power consumption, wiring complexity, thermal load and communication bandwidth. High-dimensional multimodal neural networks, dense point-cloud processing, BEV or occupancy construction and large-scale factor graph optimization can introduce substantial computational and memory burdens. In addition, temporal synchronization, extrinsic calibration, data buffering, queue management and multi-threaded scheduling increase system integration complexity. These issues are particularly critical for small UAVs, embedded mobile robots, inspection platforms and microrobots, where payload capacity and onboard computing resources are limited.

Fusion should therefore activate and process modalities according to the current task and sensor health. Sparse front ends, local maps, keyframes, sliding windows, lightweight networks, and adaptive outlier management can preserve critical constraints without running every sensor and model at full capacity.

### 5.6. Summary

Multimodal fusion improves continuity only when each modality contributes reliable, well-calibrated constraints. For resource-constrained platforms, the central trade-off is between added robustness and the sensor, computation, calibration, and power costs required to obtain it.

## 6. Edge Intelligence and Deployment of Resource-Constrained Autonomous Systems

For resource-constrained systems, offline accuracy is insufficient: perception, estimation, and fusion must execute onboard with bounded latency, power, memory, payload, thermal load, and communication. Edge deployment therefore links algorithm design to sustained closed-loop operation.

### 6.1. Deployment Constraints of Resource-Constrained Autonomous Systems

In this survey, “resource-constrained” is used as an operational category rather than a general platform label. A platform is treated as resource-constrained when onboard limits in computation, memory, power, payload, thermal capacity, or communication bandwidth materially affect algorithm selection, sensor configuration, or real-time execution. Because these limits differ across micro aerial vehicles, small UAVs, mobile robots, and AGVs, the relevant constraint should be reported together with the task and hardware configuration.

Constraints arise from limited compute, memory, storage, power, payload, cooling, and communication, together with strict real-time requirements. High-resolution images, point clouds, BEV features, and maps consume memory rapidly, while unreliable networks require continued onboard autonomy.

Consequently, intelligent navigation deployment on resource-constrained platforms should be designed from a system-level perspective. Perception models should be lightweight, state estimation should be sparsified and windowed, multimodal fusion should select only task-relevant sensors, map representations should be localized and hierarchical, and system scheduling should prioritize safety-critical modules. The goal of edge intelligence is not merely to compress models, but to establish an acceptable balance among accuracy, robustness, real-time performance and power consumption.

### 6.2. Lightweight Perception Networks and Model Compression

Deep detection, segmentation, BEV, and occupancy models are often too large or slow for sustained onboard inference. Lightweight architectures and compression are therefore central to edge perception.

MobileNet, ShuffleNet, EfficientNet, compact YOLO/Transformer variants, and related designs reduce computation through efficient convolutions, feature reuse, and simplified heads [140,141]. Pruning removes redundant structure, quantization reduces precision, distillation transfers knowledge to smaller models, and architecture search targets hardware-efficient designs.

For navigation tasks, lightweight design should be task-oriented. Autonomous systems do not always require complete semantic understanding across all object categories. Instead, they are more concerned with information directly related to motion safety and task execution, such as obstacles, traversable regions, dynamic objects, landing areas and operation targets. Therefore, edge perception models can prioritize key categories and critical spatial regions rather than pursuing comprehensive scene understanding. For BEV and occupancy representations, local BEV modeling, sparse voxel representation, and high-resolution modeling of key regions can be adopted to reduce computational and storage burdens.

It should be noted that lightweight design is not equivalent to simply reducing model size. Excessive compression may cause missed detection of small objects, inaccurate boundary segmentation and unstable recognition of dynamic targets, which may further affect downstream state estimation and path planning. Therefore, perception model optimization for resource-constrained platforms should jointly evaluate accuracy, latency, power consumption and closed-loop navigation performance, rather than relying only on parameter counts or FLOPs.

### 6.3. Efficient State Estimation and Lightweight SLAM/VIO

Compared with deep perception networks, state estimation and SLAM/VIO usually have stronger real-time requirements. For UAVs and high-speed mobile platforms, state estimation latency directly affects control stability. For mobile robots and AGVs, localization drift or mapping errors may degrade path planning and task execution. Therefore, state estimation on resource-constrained platforms must be accurate, stable, high-frequency, and capable of long-term operation.

VIO, LIO, and SLAM limit cost through sparse features, keyframes, sliding windows, local maps, marginalization, voxel filtering, and local point-cloud matching. These mechanisms avoid dense global optimization while retaining recent navigation constraints.

Learning-enhanced state estimation also needs to be lightweight when deployed at the edge. Neural noise prediction, learned features, dynamic object filtering, and observation quality assessment can improve robustness, but they should not significantly increase state estimation latency. A more suitable strategy for resource-constrained platforms is to use learning modules as auxiliary components within traditional filtering or optimization frameworks. For example, learning modules can predict observation confidence, reject dynamic features, adjust noise weights or assist loop closure detection, rather than completely replacing geometric estimation frameworks. This design can exploit the adaptability of learning-based methods while preserving the stability and interpretability of classical state estimation.

Overall, the key to efficient state estimation lies in sparsification, localization and task-driven computation. The system does not need to maintain a complete global map at every moment, nor does it need to globally optimize all historical states. Instead, it should dynamically select keyframes, local maps and optimization windows according to onboard computational resources and task requirements. Future lightweight SLAM/VIO systems are expected to focus on low-power visual front ends, sparse factor graph optimization, adaptive keyframe selection, lightweight loop closure detection and hardware-aware acceleration.

### 6.4. Hardware Acceleration and Real-Time System Integration

Edge navigation is a scheduling and integration problem as well as a compression problem. Acquisition, preprocessing, perception, estimation, mapping, planning, control, and fault monitoring run asynchronously and can create queues or accumulated latency even when each module is individually fast.

GPUs accelerate parallel inference, NPUs provide low-power neural execution, FPGAs support customized low-latency pipelines, and DSPs/MCUs handle preprocessing, filtering, or control [142]. ONNX/TensorRT conversion, quantization, operator optimization, and parallel matrix or point-cloud processing can further reduce latency.

Reliable integration requires synchronized timestamps, stable calibration and queues, priority for estimation and control, watchdogs, anomaly detection, and degradation modes for model timeout, GNSS loss, visual failure, or LiDAR degeneracy.

Long-term onboard reliability therefore depends on hardware–software co-design, real-time scheduling, energy-aware heterogeneous computing, and careful use of edge–cloud updates.

Published deployment studies show that resource constraints vary across platforms and evaluation modes [143]. Table 6 summarizes representative evidence from UAV perception and control, embedded visual-inertial odometry, monocular depth estimation, and mobile-robot SLAM. Onboard closed-loop execution, embedded replay, and offboard computation are distinguished because they impose different latency, power, payload, and communication requirements.

### 6.5. Summary

Edge deployment is a system-integration problem: model accuracy, estimator latency, memory, power, scheduling, and failure handling must be evaluated together on the target hardware. Compression is useful only when it preserves the perception and estimation functions required for safe closed-loop operation.

## 7. Datasets, Evaluation Metrics and Benchmark Platforms

Evaluation must connect algorithmic accuracy with real deployment conditions. This section reviews datasets and metrics for perception, state estimation, fusion robustness, latency, memory, and power, and examines whether current benchmarks represent resource-constrained operation.

### 7.1. Perception-Related Datasets

Autonomous-driving perception datasets differ substantially in sensor configuration and should not be treated as if they shared one complete sensor suite. KITTI provides calibrated camera and Velodyne LiDAR data, with GPS/IMU measurements available in the raw recordings. nuScenes includes six cameras, one LiDAR, five radars, GPS, and IMU, with synchronized annotated keyframes. The Waymo Open Perception Dataset provides synchronized data from five cameras and five LiDARs together with calibrations, vehicle poses, and task-specific labels. Argoverse 1 provides ring and stereo cameras, two LiDARs, six-degree-of-freedom vehicle poses, tracking annotations, and high-definition maps. These vehicle-scale benchmarks support perception and fusion research, but their sensor payloads and collection platforms are not representative of many small UAVs or lightweight mobile robots.

Indoor RGB-D benchmarks also differ in acquisition format and reference information. ScanNet contains RGB-D video sequences with reconstructed camera poses, surface meshes, and instance-level semantic annotations. SUN RGB-D consists primarily of individual RGB-D scene images collected with several camera types and provides 2D polygons, 3D bounding boxes, and room-layout annotations; it is not a continuous navigation sequence. Matterport3D provides building-scale panoramic RGB-D observations, camera poses, reconstructed meshes, and semantic annotations. These datasets support indoor perception and semantic mapping, but their reconstructed poses or static-scene annotations should not be interpreted as synchronized ground truth for long-duration robot operation.

In recent years, BEV and occupancy datasets have received increasing attention. Unlike conventional detection datasets that mainly focus on object bounding boxes, occupancy-oriented datasets emphasize spatial occupancy, semantic occupancy and 3D environmental representation. These tasks are more directly related to path planning and obstacle avoidance. For resource-constrained autonomous systems, occupancy datasets are particularly valuable because they can evaluate whether an algorithm understands the traversability of 3D space rather than merely recognizing several predefined object categories. However, most existing occupancy datasets are still designed for autonomous driving scenarios. Benchmarks targeting low-altitude UAVs, indoor mobile robots and small embedded platforms remain relatively insufficient.

### 7.2. State Estimation and SLAM Datasets

State-estimation benchmarks must be interpreted together with their synchronization and ground-truth coverage. EuRoC MAV provides hardware-synchronized stereo grayscale images at 20 Hz and IMU measurements at 200 Hz. Its machine-hall sequences use Leica laser-tracker position reference, whereas the Vicon-room sequences provide motion-capture poses. TUM VI is not an RGB-D dataset: it provides hardware-synchronized stereo fisheye grayscale images at 20 Hz and IMU measurements at 200 Hz across indoor and outdoor sequences. Motion-capture poses are available for complete room trajectories but only for the start and end segments of corridor, hall, outdoor, and slide sequences. KITTI Odometry contains 22 outdoor driving sequences with stereo images and Velodyne data; ground-truth trajectories are public for training sequences 00–10 and withheld for test sequences 11–21.

State-estimation benchmarks must be interpreted together with their synchronization and ground-truth coverage. Table 7 summarizes the benchmarks discussed in this section—EuRoC MAV, TUM VI, KITTI Odometry, UrbanNav, and UZH-FPV—together with their reference sources and coverage limits; the following paragraphs highlight where that coverage constrains what a benchmark can actually validate.For navigation involving GNSS, UrbanNav provides raw multi-constellation GNSS observations and, depending on the release and route, IMU, camera, and LiDAR measurements in dense urban environments. The records are accompanied by reference trajectories from a NovAtel SPAN-CPT GNSS/INS system. UrbanNav is therefore useful for GNSS-degraded positioning and multisensor localization under blockage, multipath, and non-line-of-sight reception, although it remains primarily a ground-vehicle benchmark. High-quality multisensor datasets with externally measured reference trajectories for low-altitude UAVs, field robots, and agricultural robots remain limited.

UZH-FPV is a real high-speed drone-racing dataset recorded indoors and outdoors. It provides conventional camera and IMU data, event-camera measurements, calibration files, and Leica laser-tracker reference poses, although ground truth is withheld for some benchmark sequences. TartanAir is a synthetic dataset whose original release provides temporally aligned stereo RGB images, depth, semantic segmentation, optical flow, camera poses, and LiDAR point clouds. AirSim and Flightmare, in contrast, are configurable simulation platforms rather than fixed datasets: their sensor configurations, sampling, scenes, and trajectories depend on the experiment. They can expose simulator-state reference data and support repeatable testing, but results remain sensitive to the selected physics, rendering, sensor-noise, and timing configurations and do not by themselves demonstrate real-platform deployment.

### 7.3. Evaluation Metrics

Different modules require different evaluation metrics. For perception tasks, 2D and 3D object detection commonly use average precision (AP), mean average precision (mAP) and nuScenes detection score (NDS), while semantic segmentation is typically evaluated using mean intersection over union (mIoU) and pixel accuracy. BEV and occupancy prediction tasks often adopt IoU, mIoU, RayIoU or voxel-level accuracy. These metrics can quantify detection and segmentation performance on offline datasets, but they do not necessarily correspond directly to navigation safety. For example, a model with high mAP may still miss small objects, distant obstacles or dynamic targets, which may lead to unsafe obstacle avoidance behavior.

For state estimation and SLAM, absolute trajectory error (ATE) and relative pose error (RPE) are different error formulations, whereas root mean square error (RMSE), mean, median, standard deviation, and maximum are summary statistics applied to a selected error [163]. ATE evaluates global trajectory consistency by comparing corresponding estimated and reference poses, while RPE evaluates local motion error or drift over a specified pose interval. Drift rate can further express accumulated translational or rotational error relative to traveled distance or elapsed time. Consequently, reporting only an ATE or RPE value is insufficient unless the trajectory association, alignment, error component, interval, and summary statistic are also specified.

Reproducible trajectory evaluation should report timestamp association or interpolation, common time span, frames, units, pose convention, and whether alignment is absent, origin-only, SE(3), or Sim(3). Scale correction should be limited to systems with unobservable scale. RPE requires the interval, unit, pair selection, and translational or rotational component. Reports should also include failures, truncated runs, relocalization, valid coverage, degradation continuity, recovery, loop closure, and uncertainty consistency.

For edge deployment, evaluation metrics should include frames per second (FPS), latency, model size, number of parameters, floating-point operations (FLOPs), memory usage, CPU/GPU/NPU utilization, and power consumption. Resource-constrained autonomous systems should pay particular attention to end-to-end latency rather than only the inference time of an individual model. A perception model with low inference latency does not necessarily guarantee a low-latency navigation loop, because image preprocessing, data transmission, state estimation, map updating, planning, and control can all introduce additional delays. Therefore, system-level evaluation should jointly consider algorithmic accuracy, inference latency, power consumption, memory usage, and closed-loop task performance.

### 7.4. Limitations of Current Benchmarks

Current benchmarks usually separate perception, estimation, deployment, and closed-loop performance; cover limited long-term environmental and calibration changes; and omit power, temperature, utilization, or hardware latency. Simulation is controllable but cannot fully reproduce sensor noise, actuation, or timing.

Therefore, future evaluation systems need to shift from offline algorithmic metrics to real system-level metrics. For resource-constrained autonomous systems, a truly valuable benchmark should answer three key questions: whether the algorithm is accurate, whether the system is real-time, and whether the platform is reliable. Only by jointly evaluating perception accuracy, state estimation error, multimodal robustness, edge resource consumption and closed-loop navigation performance can the practical value of intelligent navigation technologies be objectively reflected.

## 8. Conclusions

Evidence synthesized from the reviewed studies indicates that reliable autonomous navigation on resource-constrained platforms requires coordinated consideration of perception, state estimation, sensor fusion, and embedded execution. The reported results also reveal recurring trade-offs: improvements in accuracy or robustness often require additional sensing, computation, memory, calibration, or power. These findings are supported by the representative studies reviewed in this survey, although their numerical results are not directly comparable because the platforms, datasets, tasks, and evaluation protocols differ.

Based on this synthesis, we recommend that system designers select sensing and fusion strategies according to the target platform, operating environment, and expected failure conditions. Evaluations should report navigation performance together with deployment indicators, including hardware configuration, runtime, memory use, power consumption, and valid trajectory coverage. These statements are engineering recommendations derived from the reviewed evidence rather than universally validated design rules.

Several propositions remain open research questions. In particular, whether uncertainty-aware adaptive fusion and lightweight multimodal models can improve robustness under sensor degradation and environmental change without imposing unacceptable computational costs requires further validation. Future studies should examine these hypotheses through standardized cross-dataset evaluation and closed-loop experiments on real hardware.

## Figures and Tables

**Table 1:** Scope comparison of navigation reviews for resource-constrained autonomous systems.

Ref.	Year	Focus	Platform	Perception	State Est.	Fusion	Planning	Resources	Datasets	Main Difference
Meyer & Filliat [1]	2003	Mapping; planning	Mobile robots	○	○	—	✓	—	—	Mapping/planning
Güzel [2]	2013	Vision; mapless navigation	Autonomous vehicles	✓	○	—	○	—	—	Vision/mapless navigation
Pandey et al. [3]	2017	Navigation; avoidance	Mobile robots	○	—	—	✓	—	—	Classical avoidance
Lai et al. [4]	2025 online; 2026 issue	AUV planning	AUVs	—	—	○	✓	○	—	AUV planning
Alatise & Hancke [5]	2020	AMR fusion	Mobile robots	○	✓	✓	○	—	—	Limited deployment
Panigrahi & Bisoy [6]	2021	Localization	Mobile robots	—	✓	○	—	—	○	Localization focus
Niloy et al. [7]	2021	AMR design/control	Indoor AMRs	○	✓	○	✓	—	—	Indoor AMRs
Sánchez-Ibáñez et al. [8]	2021	Path planning	Mobile robots	—	—	○	✓	○	○	Planning focus
Wijayathunga et al. [9]	2023	Scene understanding	Ground robots	✓	○	✓	✓	—	○	Outdoor scenes
Nahavandi et al. [10]	2025	Autonomous navigation	Mobile robots	✓	✓	✓	✓	○	○	Limited resource focus
This survey	2026	Perception–deployment loop	Constrained systems	✓	✓	✓	✓	✓	✓	Resource-aware integration

Note: ✓ indicates primary coverage, ○ partial coverage, and—no explicit coverage. The comparison concerns thematic scope rather than methodological quality.

**Table 2:** Analytical dimensions of intelligent navigation for resource-constrained systems.

Dimension	Core Question	Representative Methods	Main Challenges	Section
Intelligent perception	How to represent the environment	Detection/segmentation; 2D–3D fusion; BEV; occupancy prediction	Dynamics; occlusion; geometry; real-time execution	3
Learning-enhanced state estimation	How to estimate state and uncertainty	Adaptive filtering; KalmanNet; differentiable filters; VIO/SLAM	Noise mismatch; drift; nonlinear motion; interpretability	4
Multimodal fusion	How to integrate heterogeneous sensors	Visual–inertial/GNSS; LiDAR–inertial; factor graphs	Calibration; synchronization; sensor degradation	5
Edge deployment	How to achieve real-time onboard operation	Pruning; quantization; TensorRT; sliding windows; lightweight VIO/SLAM	Power; latency; memory; reliability	6

**Table 3:** Perception tasks, outputs, and navigation uses.

Perception Task	Output	Navigation Use
Object detection	2D boxes; classes; confidence	Obstacle, landmark, and target detection/tracking
Semantic segmentation	Pixel labels; region masks	Traversability, boundaries, and scene/risk understanding
Instance segmentation	Instance masks and identities	Object separation, tracking, and target selection
Depth estimation	Depth maps; local geometry	Collision checking, free space, and local mapping
Traversable-area recognition	Free-space masks; traversable regions	Local planning, avoidance, and motion safety
Dynamic object perception	Motion states; trajectories; risk cues	Motion prediction and dynamic obstacle avoidance

**Table 4:** Complementary characteristics of navigation sensors.

Sensor	Information	Strengths	Degradation	Navigation Use
Camera	Appearance, semantics, and features	Low-cost; compact; semantically rich	Poor lighting; blur; occlusion; weak texture; weather	Visual odometry; detection; segmentation; traversability
IMU	Acceleration and angular rate	High-rate; low-latency; low-power	Bias; vibration; temperature; integration drift	Motion propagation; attitude; preintegration
GNSS	Global position and velocity	Global reference outdoors	Blockage; multipath; NLOS; indoor loss	Global localization and drift correction
LiDAR	3D range and geometry	Accurate geometry; illumination-independent	Weather; reflections; sparsity; degeneracy	Odometry; mapping; obstacle detection
Radar	Range, velocity, and coarse structure	Robust in darkness and adverse weather	Low angular resolution; sparse data; weak semantics	Adverse-weather sensing; dynamic objects
UWB	Range or indoor position	Low-power indoor absolute constraints	NLOS; anchor occlusion; infrastructure dependence	Indoor and warehouse localization

**Table 6:** Representative embedded-deployment evidence for resource-constrained navigation.

Study	Platform/Task	Input	Hardware	Accuracy/Quality	Latency/Rate	Power/Memory	Evaluation
Anwar and Raychowdhury [144]	Small UAV; DRL hallway navigation	RGB, 227 × 227	Core i7 + GTX 1080	Mean safe flight 15.6 m (baseline 16.1 m)	1.8× lower latency than baseline	0.27× energy; 0.48× GPU load; memory NR	Real UAV; offboard compute
Albanese et al. [145]	Lightweight UAV; landing-pad/person detection	Vision; LeNet-5/MobileNetV2	Raspberry Pi 3/4 + Movidius NCS2	Feasibility shown; aggregate accuracy NR	Near real time; aggregate latency NR	~10× less energy than cloud; memory NR	Onboard closed loop
Wofk et al. [146]	Embedded monocular depth	RGB, 224 × 224; NYU Depth v2	Jetson TX2	RMSE 0.604 m; δ1 0.771; 0.37 GMAC	GPU: 5.6 ms (178 fps); CPU: 37 ms (27 fps)	Active power <10 W; memory NR	Board benchmark; no closed loop
Jeon et al. [147]	MAV; VIO benchmark	D435i stereo/RGB, 640 × 480 at 30 Hz; IMU 100 Hz	TX2; Xavier NX; AGX Xavier	ATE RMSE 0.05–2.74 m across successful runs	Algorithm-specific real-time rates	8–32 GB; 7.5–30 W modes	Embedded replay of flight data
Peng et al. [148]	Mobile robot; GPU ORB-SLAM2	TUM RGB-D; Logitech C920	Jetson Xavier, TX2, and Nano	Trajectory RMSE comparable to CPU baseline	Platform- and sequence-specific frame rates	Power measured; TX2 most efficient; memory NR	Dataset replay + robot test

Note: NR denotes not reported. Values are not directly comparable because tasks, datasets, inputs, hardware, power modes, and evaluation protocols differ.

**Table 7:** Characteristics of representative datasets and simulation platforms.

Dataset/Platform	Environment	Sensors/Observations	Synchronization/Reference	Typical Tasks	Limitations
KITTI [149]	Urban/suburban driving	Stereo cameras; LiDAR; raw GPS/IMU	Calibrated recordings; benchmark-dependent annotations and OXTS poses	Detection; stereo; flow; odometry	Vehicle-scale payload; no radar; varying subsets
nuScenes [150]	Boston/Singapore driving	6 cameras; LiDAR; 5 radars; GPS; IMU	Synchronized 2-Hz keyframes; 3D boxes, maps, and ego poses	3D detection; tracking; BEV; fusion	Vehicle-scale suite; annotations slower than raw sweeps
Waymo Open Perception [151]	Multi-city driving	5 cameras; 5 LiDARs	Synchronized/calibrated data; poses and 2D/3D labels	Detection; tracking; segmentation	No raw radar/GNSS/IMU stream; vehicle scale
Argoverse 1 [152]	Pittsburgh/Miami driving	9 cameras; 2 LiDARs; HD maps	Per-log calibration, 6-DoF poses, and tracking/forecasting labels	Tracking; forecasting; map-aware perception	Driving-specific platform and tasks
ScanNet [153]	Static indoor scans	RGB-D video	Reconstructed poses, meshes, and semantic labels; no external trajectory GT	3D segmentation; reconstruction; semantic mapping	Static scenes; reconstructed rather than operational poses
SUN RGB-D [154]	Static indoor scenes	Single RGB-D images	N/A; 2D polygons, 3D boxes, and room layouts	2D/3D detection; scene understanding	No temporal navigation sequences
Matterport3D [155]	90 indoor buildings	Panoramic RGB-D; 3D meshes	N/A; poses, meshes, and semantic labels	Reconstruction; semantic mapping; embodied navigation	Static panoramas; no real-time resource record
EuRoC MAV [156]	Machine halls/Vicon rooms; MAV	Stereo 20 Hz; IMU 200 Hz	Hardware synchronized; Leica or Vicon reference	VIO; SLAM; state estimation	Short indoor sequences; reference timing varies
TUM VI [157]	Handheld indoor/outdoor	Stereo fisheye 20 Hz; IMU 200 Hz	Hardware synchronized; 120-Hz mocap in rooms and at other sequence endpoints	VIO; calibration	No depth; full GT unavailable for most non-room sequences
KITTI Odometry	Road driving	Stereo cameras; LiDAR; OXTS GPS/IMU	Calibrated streams; public GT for 0–10	Visual/LiDAR odometry; SLAM	Road motion; limited small-robot relevance
UrbanNav [158]	Dense urban canyons; vehicle	Multi-GNSS; IMU; camera/LiDAR by subset	Timestamped data; SPAN-CPT GNSS/INS reference	GNSS/INS fusion; localization; SLAM	Sensor suite varies; mainly vehicle data
UZH-FPV [159]	Drone racing	Images/IMU; DAVIS events/images; FPV RGB	Timestamps/calibration; Leica poses; some GT withheld	High-speed VIO; visual/event state estimation	Specialized trajectories; partial public GT
TartanAir [160]	Synthetic indoor/outdoor	Stereo RGB; depth; segmentation; flow; poses; LiDAR	Common simulation timeline; exact poses and dense labels	SLAM; depth; flow; navigation	Synthetic appearance, motion, and noise
AirSim [161]	Configurable simulation	Cameras; depth; segmentation; flow; IMU; GPS; LiDAR	Common API time; simulator state and dense reference	Perception; control; navigation; RL	Not fixed; fidelity depends on configuration
Flightmare [162]	Quadrotor simulation	Rendering; cameras; point clouds; vehicle state	Controlled rendering/physics; exact simulated state	Quadrotor control; perception; RL	Not fixed; sim-to-real/configuration dependence

Note: GT denotes ground truth. Reference data may include external trajectories, reconstructed poses, annotations, or simulator states. N/A indicates that synchronization is inapplicable to static data; sensors may vary by release or sequence.

## Data Availability

No new data were created or analyzed in this study. Data sharing is not applicable to this article.

## Abbreviations

The following abbreviations are used in this manuscript:

UAV	Unmanned Aerial Vehicle
BEV	Bird’s Eye View
AGV	Automated Guided Vehicle
SLAM	Simultaneous Localization and Mapping
GNSS	Global Navigation Satellite System
VIO	Visual-Inertial Odometry
FPV	First Person View
IMU	Inertial Measurement Unit
UWB	Ultra-Wideband
MEMS	Micro-Electro-Mechanical System
AI	Artificial Intelligence
CPU	Central Processing Unit
GPU	Graphics Processing Unit
